# SeDeM expert system with I-optimal mixture design for oral multiparticulate drug delivery: An encapsulated floating minitablets of loxoprofen Na and its *in silico* physiologically based pharmacokinetic modeling

**DOI:** 10.3389/fphar.2023.1066018

**Published:** 2023-03-03

**Authors:** Rauf-ur-Rehman, Muhammad Harris Shoaib, Farrukh Rafiq Ahmed, Rabia Ismail Yousuf, Fahad Siddiqui, Muhammad Talha Saleem, Faaiza Qazi, Momina Zarish Khan, Asma Irshad, Lubna Bashir, Shazia Naz, Muhammad Farooq, Zafar Alam Mahmood

**Affiliations:** ^1^ Department of Pharmaceutics, Faculty of Pharmacy and Pharmaceutical Sciences, University of Karachi, Karachi, Sindh, Pakistan; ^2^ Department of Pharmaceutics, Faculty of Pharmacy, Federal Urdu University of Arts, Science and Technology, Karachi, Pakistan

**Keywords:** multiparticulate drug delivery system, SeDeM expert tool, loxoprofen Na, *in silico* PBPK, extended release minitablets

## Abstract

**Introduction:** A SeDeM expert tool-driven I-optimal mixture design has been used to develop a directly compressible multiparticulate based extended release minitablets for gastro-retentive drug delivery systems using loxoprofen sodium as a model drug.

**Methods:** Powder blends were subjected to stress drug-excipient compatibility studies using FTIR, thermogravimetric analysis, and DSC. SeDeM diagram expert tool was utilized to assess the suitability of the drug and excipients for direct compression. The formulations were designed using an I-optimal mixture design with proportions of methocel K100M, ethocel 10P and NaHCO_3_ as variables. Powder was compressed into minitablets and encapsulated. After physicochemical evaluation lag-time, floating time, and drug release were studied. Heckel analysis for yield pressure and accelerated stability studies were performed as per ICH guidelines. The *in silico* PBPK Advanced Compartmental and Transit model of GastroPlus™ was used for predicting *in vivo* pharmacokinetic parameters.

**Results:** Drug release follows first-order kinetics with fickian diffusion as the main mechanism for most of the formulations; however, a few formulations followed anomalous transport as the mechanism of drug release. The *in-silico*-based pharmacokinetic revealed relative bioavailability of 97.0%.

**Discussion:** SeDeM expert system effectively used in QbD based development of encapsulated multiparticulates for once daily administration of loxoprofen sodium having predictable *in-vivo* bioavailability.

## Introduction

Gastroretentive drug delivery systems (GRDDS) are designed to retain themselves in the stomach after oral administration for an extended period, thereby releasing the drugs that are subsequently absorbed through the stomach and duodenum (Eberle et al., 2014). GRDDS are favorable for drugs with a narrow absorption window, high solubility in acidic pH, shorter half-life, and/or promote local activity in the stomach and duodenum (Jagdale et al., 2013). Various approaches have been devised to attain gastric retention of the dosage forms. These include floating drug delivery systems, mucoadhesive systems, high-density systems, and expandable and swelling systems (Pawar et al., 2011; Qin et al., 2018).

Floating drug delivery systems have certain advantages in comparison to others which include the lesser impact on gastric motility and reduced mucosal damage (Arora et al., 2005; Bardonnet et al., 2006). Moreover, in contrast, to single-unit floating systems, multiple-unit floating systems (granules, pellets, and minitablets) offer less variability in drug release and reduced chances for gastric emptying of the entire multi-unit system (Meka et al., 2009; Louis et al., 2020a). Additionally, the floating systems comprising directly compressible mini-tablets offer a significant advantage over pellets or granules based systems in terms of ease of manufacturing and processing controls (Zhu et al., 2014; Lopes et al., 2016). Mini-tablets are also a suitable dosage form for pediatric and elderly patients (Aleksovski et al., 2015).

SeDeM expert system is an innovative galenical tool for evaluating the suitability of the pharmaceutical ingredients to be converted into tablet dosage forms by means of direct compression technique (Aguilar-Díaz et al., 2009; Aguilar-Díaz et al., 2014). It provides complete information on the powder characteristics in the form of 12 parameters that provide guidance on powder materials and their deficiencies to be worked on to make the blends suitable for direct compression (Suñé-Negre et al., 2008; Campiñez et al., 2016).

Loxoprofen sodium is a non-steroidal anti-inflammatory drug (NSAID) that selectively inhibits the cyclooxygenase (COX) enzyme. It has a prominent analgesic effect in different chronic disorders such as arthritis, osteoarthritis, rheumatoid arthritis, lumbago, cervical spondylosis, joint disorders, musculoskeletal, toothache, and post-operative procedures also show antipyretic activities (Ryu and Shin, 2011; Nanthakumar et al., 2016). It is also used to treat benign prostatic hyperplasia (BPH) with complaints of nocturia (Shin et al., 2011). Loxoproen sodium is a weakly acidic prodrug of propionic acid derivative. It is water-soluble drug with a shorter half-life, absorbed in free acid form, and has less gastric irritation and toxicity therefore does not cause direct adverse reactions to the gastric mucosa, (Sakamoto and Soen, 2011; Zaman et al., 2013a). A double-blind controlled study in healthy Japanese volunteers revealed that subjects on loxoprofen had a significantly lower rate of gastric injury than those on diclofenac (Yanagawa et al., 1992). In Asian patients, only 0.24% reported gastrointestinal bleeding and required hospitalization and were considered safe compared to other non-selective NSAIDs such as indomethacin and diclofenac (Waikakul and Waikakul, 1999). Furthermore, it has a relatively lower membrane permeability and cytotoxic effect on gastric mucosal cells as compared to other drugs of this class (Yamakawa et al., 2010a). Because of the shorter half-life of about (2–4 h), frequent administration is required to maintain the concentration in the therapeutic window (Cho et al., 2006). Such problems can be minimized by developing a multi-unit floating gastroretentive drug delivery system of loxoprofen sodium, which decreases the dosing frequency and maintains the plasma drug concentration for a prolonged period (Ajay et al., 2010; Krunal et al., 2011).

Physiological-Based Pharmacokinetics’ (PBPK) modeling and simulation is an *in silico* computational tool for the evaluation and estimation of the *in-vivo* performance of the drugs based on the information gathered regarding the biopharmaceutics and pharmacokinetic profile of the given drug (Pelkonen et al., 2011; Jones et al., 2015; Cvijic et al., 2018a). PBPK modeling and simulation is currently becoming an essential part of drug development and discovery (Jones et al., 2015).

In this study, a facile and directly compressible tableting approach using SeDeM expert tool-driven design of experiments (DoE) has been employed to develop a multiple-unit floating gastroretentive drug delivery system of loxoprofen sodium. Moreover, mechanistic “physiologically based pharmacokinetics” (PBPK) modeling has been utilized to simulate and predict the *in-vivo* performance of the developed system in comparison to the immediate release profile of the drug.

The reason for using Loxoprofen sodium as a model drug for gastroretentive floating system is because multiple-units floating system offers advantages over single units such as lower toxicity risk (due to a lower risk of dose dumping), reduced dependency on gastric emptying (which leads to a lesser degree of inter and intra-individual variability), avoidance of the all-or-none effect (the failure of individual units does not compromise the entire system), and greater dispersion throughout the digestive tract (which lowers the risk of local high concentrations, minimizing local irritation and allowing for greater drug protection) (Lopes et al., 2016). The developed formulations have been characterized and evaluated for their drug-release and floating characteristics.

## Materials and methods

### Materials

Loxoprofen sodium (99.4% w/w) originally synthesized by Daiichi Sankyo ProPharma Co. Ltd. (Tokyo, Japan) was received as a gift sample from Hilton Pharmaceuticals (Karachi, Pakistan). Hydroxypropyl methylcellulose (Methocel K4M, K15M, and K100M), and Ethylcellulose (Ethocel 10 premium) were generously provided by Colorcon limited (Kent, United Kingdom). Sodium bicarbonate and magnesium stearate were purchased from Sigma Aldrich (Darmstadt, Germany). Hydrochloric acid (37% w/v), potassium dihydrogen phosphate (≥99.5% w/w), sodium hydroxide (≥99% w/w), acetonitrile (≥99.99% w/w), and acetone (≥99.8% w/w) were all purchased from Merck (Darmstadt, Germany). Entire chemicals and solvents used in the study were of analytical grade.

### SeDeM based characterization of excipients and API

Loxoprofen sodium and other excipient materials (methocel K100M, ethocel 10P and sodium bicarbonate) were assessed for their suitability for direct compressibility by SeDeM diagram expert tool. For this purpose, various compressional and flow characteristics of the powder materials were experimentally evaluated, and thereby the 12 SeDeM parameter values (bulk and tapped density, interparticle porosity, Carr’s index, and Hausner’s ratio, cohesion index, angle of repose, flowability, loss-on-drying, hygroscopicity, particle size below 50 µm and homogeneity index) were calculated (Aguilar-Díaz et al., 2009; Aguilar-Díaz et al., 2014) (see Table1). For experimental methodology, various compendial methods as described in the European pharmacopeia were employed with slight modifications wherever needed, and the detailed methodology is given in the supplementary section (*Methodology for SeDeM*) ***.*** All tests were performed in triplicate to reduce the chances of variation (Ahmed et al., 2019).

**TABLE 1:** SeDeM incidence parameters, parameters, limit values and conversion factor to convert each parameter into radius (*r*).

Incidence	Parameter	Symbols	Units	Equation	Acceptable range	Factor applied to value (v)	Radius values (r)
Dimensions	Bulk density	Da	g/mL	Da = Pa/Va	0–1 g/mL		0–10
	Tapped density	Dc	g/mL	Dc = Pc/Vc	0–1 g/mL	10 *v*	0–10
Compressibility	Inter-particle-porosity	Ie	-	Ie = Dc –Da/Dc × Da	0–1.2	10*v*/1.2	0–10
	Carr’s index	Ic	%	Ic = D_c_ –Da/Dc ×100	0%–50%	*v*/5	0–10
	Cohesion index	Icd	N	Experimental	0–200 (N)	*v*/20	0–10
Flowability/powder flow	Hausner ratio	IH	-	IH = D_c_/Da	3–1	30–10*v*/2	0–10
	Angle of repose	α	-	tα = H/R	50–0 (°)	10-*v*/5	0–10
	Powder flow	t	S	Experimental	20–0 (s)	10-*v*/2	0–10
Lubricity/stability	Loss on drying	%HR	%	Experimental	0–10 (%)	10-*v*	0–10
	Hygroscopicity	%H	%	Experimental	20–0 (%)	10-*v*/2	0–10
Lubricity/dosage	Particle <50um	%Pf	%	Experimental	50–0 (%)	10-*v*/5	0–10
	Homogeneity index	Iθ	-	Iθ = Fm/100×ᶺf m_n_	0-2×10-2	500*v*	0–10

### Drug-excipients compatibilities studies

To evaluate the drug excipient compatibility, stress stability studies were conducted on equal proportions of drug and three formulation functional excipients [Methocel K100M, Ethocel 10P and NaHCO3] (% w/w; 1:1:1:1). The physical mixture was incubated at 40°C ± 2°C and RH of 75% ± 5% for 30 days in a stability chamber (NuAire, Plymouth, MN, United States). The sample was then withdrawn and subjected to Fourier transform infrared (FTIR) spectroscopy, differential scanning calorimetry (DSC), and thermogravimetric analysis (TGA) to evaluate any incompatibility arising in form of any chemical or physical changes of the drug (ICH, 2003; Vyas et al., 2016).

#### Fourier transform infrared (FTIR) spectroscopy

Fourier transformed infrared (FTIR) spectroscopy of loxoprofen Na, Methocel K100M, Ethocel 10P and NaHCO3 was performed on Bruker Alpha E instrument (Borken, Germany). The samples were placed on the diamond attenuated total reflectance (ATR) and analyzed in the wavelength range of 4,000–500 cm-^1^.

#### Differential scanning calorimetry (DSC) and thermogravimetric analysis (TGA)

TA instrument’s SDT-650 simultaneous TGA-DSC thermal analyzer (TA instruments; Waters, DE, United States) was used for the measurement of the thermal analysis of the drug, excipients, and drug-excipient mixture in equal proportion (1:1:1:1). Around 4 mg of each sample was placed in the sample holder and empty platinum holder was used as reference. The samples were analyzed for dynamic scanning calorimetry and thermogravimetric analysis simultaneously at a rate of 10°C/min. Nitrogen gas flow was maintained at a rate of 99.98 mL/min.

### Experimental design

An I-optimal mixture design using Design Expert, ver. 10.0.1 (Stat-Ease, Minneapolis, United States) was used to prepare the experimental gastro-retentive mini-tablet formulations of loxoprofen sodium. The minimum percentages of excipients required for correction of the most deficient incidence parameter value (SeDeM expert tool) of loxoprofen sodium were calculated using the following equation.

$$CP=100-\left[\left(\left(\left(RE-R\right)/\left(RE-RP\right)\right)\right)\times 100\right]$$ (1)

Where CP = Percentage of excipient required to correct the index in the deficient material. RE = Incidence parametric value of excipients added to correct the API. Rp = Incidence, the parametric value of the subjected material to be corrected and R= (desired incidence parameter value > 5) (Suñé-Negre et al., 2008).

The experimental design and proportions of the formulation variables are given in Table 2.

**TABLE 2:** Constraints for numerical optimization for the I-optimal mixture design and final equations for the dependent variables.

Optimization constraints					Final equations
Independent variables	Levels		Optimization goal	Weightage/importance	
	Low (%)	High (%)			
Methocel K100M (X1)	34	45	in range	3	-
Ethocel 10P (X2)	0	10	in range	3	-
NaHCO_3_ (X3)	5	15	in range	3	-
Dependent variables					
Floating lag time (sec)	18	60	minimum	5	Floating lag-time = + (39.529*X1) + (101.567*X2)–(51.589*X3)
Total floating time (h)	15.5	22	maximum	4	Total floating time = +(21.05*X1) + (8.90*X2)–(141.18*X3)–(48.46*X1*X2) + (187.72*X1*X2) + (428.77*X1*X2)
Drug release 1 h (%)	13.23	28.33	minimum	3	drug release @1 h =–(789.73*X1) + (14253.10*X2) + (11402.11*X3)–(20181.09*X1*X2)–(20932.57*X1*X2)–(25512.018*X1*X2)–(2330.21*X1*X2*X3) + (10682.79*X1*X2*(X1-X2) + (17926.72*X1*X2*(X1-X3)–(25222.90*X2*X3* (X2-X3)
Drug release 12 h (%)	71.58	87.7	in range	3	drug release @12h =–(209.032*X1)–(6036.65*X2)–(2407.24*X3) +9371.45*X1*X2) + (4475.80*X1*X3) + (27922.07*X2*X3)–(37539.87*X1*X2*X3)
Drug release 24 h (%)	96.01	100.15	maximum	3	drug release @24 h = + (82.14*X1)–(2811.41*X2) + (2135.68*X3) + (4,647.22*X1*X2)–(3597.37*X1*X3) + (1853.97*X2*X3)–(926.10*X1*X2*X3)–(1790.63*X1*X2*(X1–X2)) + 1900.98*X1*X3*(X1-X3) + 4404.16*X2*X3*(X2-X3)

The final weight of minitablets was set at ∼15 mg (6 mg loxoprofen sodium). Three independent variables used in the design were “methocel K100M” in the range of 34%–45%, “ethocel 10P” in the range of 0%–10%, and “sodium bicarbonate” from 5% to 15%, whereas responses include floating lag time, total floating time, percent drug release at 1 h, 12 h and 24 h (Gong et al., 2018).

### Tableting and encapsulation

The mini-tablets were prepared by means of the direct compression technique. For this purpose, the drug and the excipients were first sieved through USP sieve no. 60. (Stavebni Stroje, Chechoslovakia)) to get uniform and sufficiently small particle size to ensure a well-mixed blend. The powdered blends were initially mixed at 2, 5, and 9 min in a tumbler-type mixer (Cube mixer, Erweka, Heusenstamm, Germany), and the tablets of 15 mg approx. Were prepared, and individual units were assayed and evaluated against a criteria of <5% RSD drug content in the individual units. The powdered materials were then mixed for 5 min (satisfactory time for mixing) (Carstensen and Carstensen, 1993; FDA, 2003). Afterward, the mixed blend was added with the required amount of magnesium stearate and again mixed for 2 min in a tumbler type mixer. The formulation blends were then compressed into tablets (∼15 mg; 6 mg loxoprofen sodium) using an eccentric-type single punch tablet machine (AMW, Pakistan) comprising of a die and flat-shaped punches with a diameter of approximately ∼3 mm and an average thickness of ∼2.15 mm. The detailed composition of the tablets is given Table 2. These mini-tablets were then enclosed in hard gelatin capsules (size “00”) as a set of 20 tablets (120 mg loxoprofen sodium) in each capsule.

### Pharmaceutical quality evaluation

The prepared gastro-retentive minitablets were subjected to various physicochemical tests to evaluate their pharmaceutical quality such as variations of tablets in terms of their weight, thickness, hardness, and friability. For this purpose, 20 mini-tablets were first selected randomly, and weight measurement was undertaken using an analytical balance (moisture balance EB-340; Shimadzu corporation, Japan). Moreover, the diameter, thickness, and hardness variation were performed using Dr. Schleuniger Pharmatron M50 multi-test instrument (SOTAX; Nordring, Aesch, Switzerland). For friability testing, a Roche-type friability tester (Erweka D22800; GmbH; Heusenstamm, Germany) was used, and a total of 10 tablets were rotated inside the drum at 25 rpm for 4 min and the percentage weight loss was calculated (Jagdale et al., 2013; Nart et al., 2017; Louis et al., 2020b). Uniformity of content was estimated with a Shimadzu LC-10AT VP with UV–visible detector (Shimadzu Corporation, Kyoto, Japan) with C-18 reverse phase column (Mediterrania Sea, 5 μm, 250 mm × 4.6 mm, Teknokroma, Spain). The details of the assay procedure are given in the proceeding sections.

### Scanning electron microscopy (SEM)

The surface morphology of the formulations was evaluated using a scanning electron microscope (JEOL JSM 6380, Japan) at an accelerating voltage of 25–30 kV and with appropriate 5,000x-8000x magnifications at room temperature. The samples were attached to aluminum stubs with double side adhesive carbon tape, gold coated (250 0A JFC-1500 JEOL), and sputter coater. SEM photographs were recorded and examined (Husain et al., 2021).

### Uniformity of content

The content uniformity of the loxoprofen mini-tablets was carried out in a mobile phase consisting of acetonitrile and water mixture (40:60) with a pH of 6.5 (adjusted using dihydrogen sodium). Prepared samples were filtered using 0.45 µm millipore filter paper. The volume injected for the assay was 10 uL. Estimation of Loxoprofen sodium content was carried out by using HPLC validated method, Analysis was performed using Shimadzu HPLC- LC-10AT (Shimadzu Corporation, Kyoto, Japan) configured with an SPD-10A UV-Visible detector (Shimadzu Corporation, Kyoto, Japan) The system was supplied an HPLC reverse phase column (Mediterranea sea C18-250 × 4.6 mm, 5 um Teknokroma Spain) (Eberle et al., 2014). Mean values and standard deviation of three readings were recorded.

### Buoyancy studies

Buoyancy studies were performed *in-vitro* on three units of each formulations (F1-F16) to estimate the “floating lag time” and “total floating time.” For this purpose, mini-tablets were dropped in 250 mL of 0.1 N HCl (pH 1.2) and maintained at 37°C. The time required for the tablets to rise at the surface after immersion was counted as “floating lag time” while the total time period for which the tablets remained afloat at the surface was counted as “total floating time” (Louis et al., 2020c).

### Swelling and erosion studies

Swelling and erosion behavior of the mini tablets were carried out in triplicate for each formulation (F1–F16). For this purpose, each unit tablet was weighed (moisture balance EB-340; Shimadzu corp., Japan) and dropped individually in a USP apparatus II (paddle assembly; Erweka DT600; Heusenstamm, Germany) containing 900 mL of 0.1 N HCl (pH 1.2) and run at 50 RPM. The sample tablets were taken out at different intervals, and the excess water was absorbed on the bloating paper. Finally, the swelling and erosion were calculated using the following equations (Chen et al., 2015).

$$\%\;swelling=W_{1}-W_{2}/w2\times 100$$ (2)

$$\%\;erosion=W_{0}-\frac{W_{2}}{w2}\times 100$$ (3)

Where W_1_ = initial weight, W_2=_ weight after swelling and W_0=_ weight after erosion.

### Drug release studies and kinetic profiling

*In-vitro* drug release profiles of each of the formulations were generated for 24 h using USP apparatus II (paddle assembly; Erweka DT-600; Heusenstamm, Germany). For this purpose, 20 minitablets encapsulated in hard shell capsule (one capsule sized “00” unit with 20 tablets containing 6 mg loxoprofen Na each; a total of 120 mg of loxoprofen Na) were dropped in the dissolution beakers containing 900 mL of 0.1 N HCl buffer (pH 1.2), and the paddle assembly was run at 50 RPM (Louis et al., 2020b). The samples (5 mL) were withdrawn at predetermined time points, and the medium was replaced with fresh buffer to maintain the sink condition. Further, the samples were filtered by a millipore filter (0.45 µm pore size) to remove suspended and insoluble components. Finally, the concentration of loxoprofen sodium was determined by a UV-visible spectrophotometer (UV-1800 Shimadzu Corporation, Kyoto, Japan) at λmax of ∼223 nm (spectra generated for each sample in the range of 200–400 nm).

The drug release data were modeled with various model dependent kinetic procedures. These included empirical (zero-order, first-order, and Higuchi kinetics) and mechanistic semi-empirical models (Hixon-Crowell and Korsmeyer-Peppas). The drug release kinetic modeling was performed in Microsoft Excel 2013 adds-in package named DD Solver (China Pharmaceutical University, Nanjing, China) (Zhang et al., 2010).

### Optimization

The optimized formulations selected based on the four responses identified in the study included 1) minimum floating lag-time (sec), 2) total floating time (h), and 3) percent drug release at 1, 12, and 24 h (Gong et al., 2018) (see Table 3).

**TABLE 3:** Composition of the formulation blends (F1-F16) of loxoprofen sodium calculated through I-optimal mixture design.

Formulation code	Methocel K100M		Ethocel 10P		NaHCO_3_		Mg stearate		Loxoprofen sodium		Net weight
	(%)	(mg)	(%)	(mg)	(%)	(mg)	(%)	(mg)	(%)	(mg)	(mg)
F1	44.5	6.67	0.0	0.00	14.6	2.18	1.0	0.15	40.0	6	15
F2	36.6	5.48	10.0	1.50	12.4	1.87	1.0	0.15	40.0	6	15
F3	45.0	6.75	4.6	0.69	9.4	1.41	1.0	0.15	40.0	6	15
F4	39.3	5.90	9.6	1.44	10.1	1.51	1.0	0.15	40.0	6	15
F5	39.2	5.88	4.9	0.73	14.9	2.23	1.0	0.15	40.0	6	15
F6	44.2	6.63	9.8	1.47	5.0	0.75	1.0	0.15	40.0	6	15
F7	44.2	6.63	9.8	1.47	5.0	0.75	1.0	0.15	40.0	6	15
F8	36.6	5.48	7.4	1.12	15.0	2.25	1.0	0.15	40.0	6	15
F9	44.5	6.67	0.0	0.00	14.6	2.18	1.0	0.15	40.0	6	15
F10	45.0	6.75	4.6	0.69	9.4	1.41	1.0	0.15	40.0	6	15
F11	34.0	5.10	10.0	1.50	15.0	2.25	1.0	0.15	40.0	6	15
F12	39.3	5.90	9.6	1.44	10.1	1.51	1.0	0.15	40.0	6	15
F13	40.8	6.12	6.4	0.96	11.8	1.77	1.0	0.15	40.0	6	15
F14	42.6	6.39	7.9	1.18	8.5	1.27	1.0	0.15	40.0	6	15
F15	43.0	6.45	3.2	0.48	12.8	1.92	1.0	0.15	40.0	6	15
F16	39.2	5.88	4.9	0.73	14.9	2.23	1.0	0.15	40.0	6	15

### Heckel analysis

Loxoprofen sodium and excipients were accurately weighed, sieved, and mixed for a few minutes by tumbling in a tumbler mixer. After mixing the excipients and API, magnesium stearate 1% was added as a lubricant and further blended for 5 min. The mixture was then filled manually into the dies, and mini-tablets were compressed at various pressures in the range of 15–148 MPa using a hydraulic tableting machine (Natoli NP-RD10, MO, United States). The compressional and ejection data was acquired through “Natoli AIM TM pro plus” software (Natoli Engineering Inc. MO, United States). The compressed mini-tablets were placed in a desiccator for 24 h over silica gel for hardening and elastic recovery. The mathematical expression of the Heckel plot equation is mentioned below**.**

$$\ln\left[\frac{1}{1}-\mathrm{pr}\right]=KP+A$$ (4)

$$\mathrm{pr}=\frac{pa}{pt}$$ (5)

$$Pa=\frac{weight\;of\;minitablet\;}{\pi r^{2}h}$$ (6)

Where p = applied pressure, “K” and “A” are slope intercepts, p_r_ (relative density), p_A_ (apparent density), and P_t_ (true density of powder blends).

### Stereomicroscopic analysis

Stereomicroscopic images of compressed loxoprofen sodium gastro-retentive floating mini tablets were carried out using a stereomicroscope (AmScope. LED digital stereomicroscope, SM-1TSZZ-144S, Amscope; Irvine, CA, United States). Three swelling dynamics at different time points (0, 1, 3, and 5 min) after contact with dissolution media (3 mL of 0.1 N HCl buffer, pH 1.2) were observed, and formulations were evaluated for any surface structure deformity and swelling in both radial and axial directions (Lazzari et al., 2018).

### Stability studies

Three different batches of the optimized formulation (F2) were subjected to accelerated stability testing (40°C/75% ± 5% RH) for up to 6 months in a stability chamber (NuAire, Plymouth, MN, United States), according to the International Conference on Harmonization of “Medicines for Human Use” (ICH) guidelines (as adopted by WHO) (Organization, 2018). Formulations were evaluated for various physical characteristics (color, shape, and morphology) and physicochemical parameters, i. e., hardness, friability, floating lag time, and total-floating time. Formulations were further evaluated for *in-vitro* drug release and content uniformity. “Minitab” statistical software, version 20 (Minitab, Pennsylvania, United States) was used to determine the shelf-life.

### *In-silico* PBPK modeling and simulation

The *in-vitro* drug release data (in 0.1 N HCl; pH 1.2) of the optimized mini-tablet formulation (F2) having once-daily dose of 120 mg was applied to *in silico* “Physiological Based Pharmacokinetics” (PBPK) modeling and simulation. These simulation studies were carried out using (ACAT) “Advanced Compartmental and Transit” model presented in GastroPlus™ software version 9.8 (Simulations Plus Inc., Lancaster, CA, United States). Various physicochemical properties of loxoprofen sodium (API) such as Log P, molecular weight (Mw), drug particle density diffusion coefficient, jejunal effective permeability, and human blood-plasma concentration ratio of loxoprofen sodium were calculated and obtained from the (ADMET)TM predictor module of the software (GastroPlusTM). Whereas other physicochemical properties such as pKa, aqueous solubility, and unbound fraction of the drug in plasma were used as reported in the literature. The description of these input parameters for the ACAT model is given in Table 4 (D.D.W.s. Laboratory, 2022; Kambayashi and Yomota, 2021; Adachi et al., 2021).

**TABLE 4:** Parameters and extracted values for “Advanced Compartmental and Transit” (ACAT) modelling in GastroPlus™.

Input biopharmaceutical and physicochemical parameters for simulation		
Parameter	Value	Source
Log P	2.99	ADMET Predictor™
pKa	4.19	Kambayashi and Yomota, 2021
Molecular Weight (g/mol)	304.3	ADMET Predictor™
Aqueous Solubility (mg/mL)	0.0268	Louis et al. (2020b)
Diffusion Coefficient (cm2/sec x 10–5)	0.75	ADMET Predictor™
Drug Particle Density (g/mL)	1.62	ADMET Predictor™
Jejunal Effective Permeability (Peff) (cm/sec x10-4)	5.31	ADMET Predictor™
Unbound Percent in Human Plasma (Fup %)	1%	Adachi et al., 2021
Human Blood to Plasma Concentration Ratio (Rbp)	0.69	ADMET Predictor™
Vc (L/kg)	0.0381	PKPlus™
K_12_ (L/h)	0.3013	PKPlus™
K_211_ (L/h)	0.0211	PKPlus™
Clearance (L/h/kg)	0.10354	PKPlus™

Since the drug reportedly follows 2-compartment pharmacokinetics, the parameter values for the 2-compartment model of loxoprofen sodium such as VC, K12, K21, and CL were used as described in the literature for the *in-vivo* pharmacokinetics of immediate-release loxoprofen sodium formulation by (Kang et al., 2011). Utilizing these literature reported values as input parameters and *in-vitro* drug release data of optimized loxoprofen sodium gastroretentive mini-tablets formulations (F2), *in-vivo* drug concentration profiles were simulated and the corresponding Cmax, T_max_, AUC_t_, AUCinf, were calculated (Cvijic et al., 2018b). Finally, the simulated pharmacokinetic profiles of the optimized formulations were compared with the *in-vivo* profile of single-dose 60 mg loxoprofen sodium following the methodology reported in the literature, and relative bioavailability for optimized formulations was calculated. The fold error (FE) and error percentage (%PE) of prediction was applied between the observed and simulated data using the following equations (Cvijic et al., 2018):

Fold Error=Observed value/predicted value (7)

$$\%PE=\left(Observed-predicted\right)/Observed*100$$ (8)

## Results

### SeDeM based characterization

**FIGURE 1:**
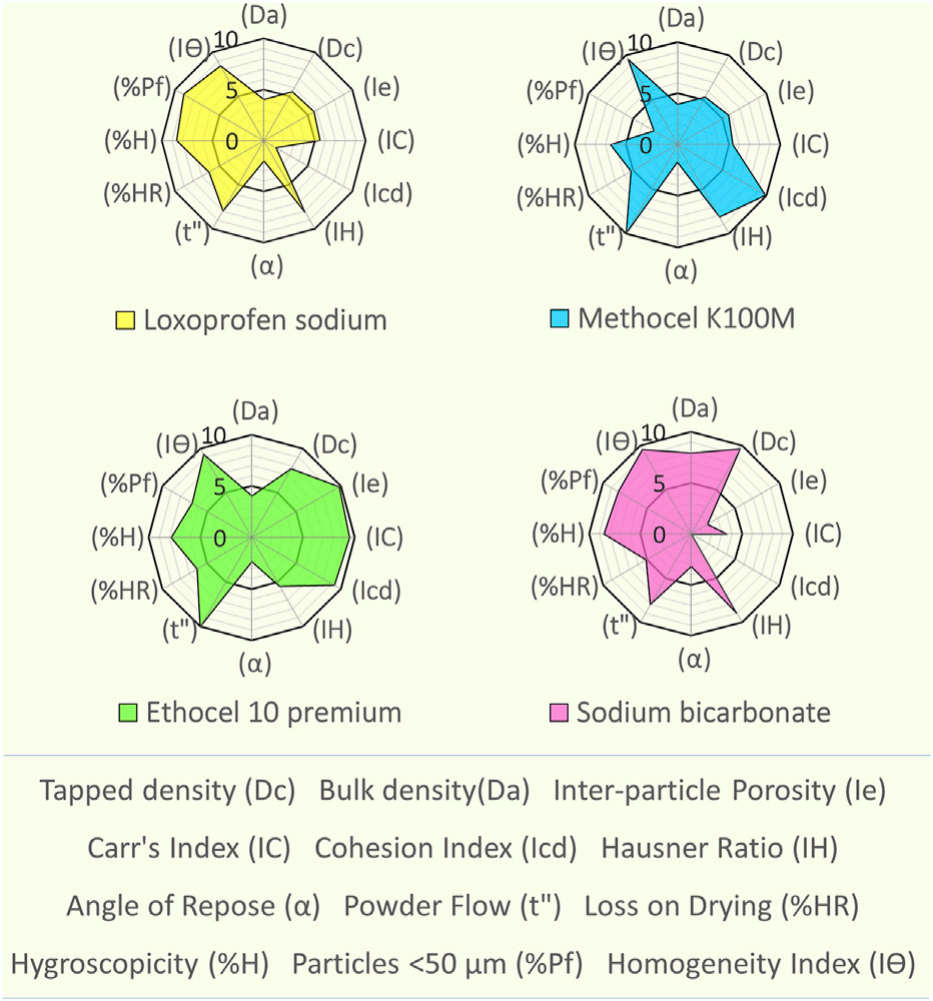
SeDeM expert tool diagram of loxoprofen sodium, methocel K100M, ethocel 10 premium and sodium bicarbonate.

The SeDeM based assessment of loxoprofen sodium and excipients was carried out (see Figure 1 and Supplementary Tables S1, S2). An assessment revealed its deficiency in terms of cohesion index (1.42) and angle of repose (2.02), which affects the hardness and flow properties of the material. However, the drug material exhibits good to excellent values for the remainder of the parameters. The SeDeM based investigation for the functional polymers (methocel K15M, K100M and ethocel 10-P) (see Figure 1 and Supplementary Tables S1, S2) The “Good Compressibility Index” (ICG) values were calculated above 5 as well, showing high suitability of all the polymers for the direct compression (see Figure 1 and Supplementary Table S2). The minimum percentages of the excipients for the correction of the API were calculated as, Methocel K15M, 28.46%; K100M, 33.3%, and Ethocel 10P as ∼15%) (see Table 2). Since sodium bicarbonate was to be used in minimal quantities (5%–15%) (see Table 2), as a functional excipient (effervescent agent), its only deficiency in respect of the “compressibility incidence” profile did not matter much in the overall quality expectations of the formulation blends.

### Excipient compatibility studies

**FIGURE 2:**
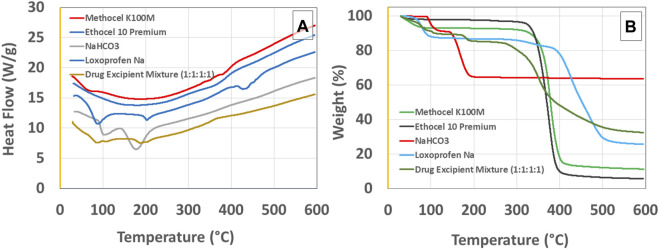
**(A)** Differential scanning calorimetry (DSC) and **(B)** thermogravimetric analysis (TGA) graphs for loxoprofen sodium, methocel K100M, ethocel 10P, sodium bicarbonate, and Drug Excipient Mixture (1:1:1:1 ratio incubated for 30 days at 40°C/75% ± 5% RH).

Loxoprofen sodium and excipient compatibility studies were carried out in accelerated (40°C/75% ± 5% RH) conditions for the formulation blends in 1:1:1:1 mixture composition for 30 days after their characterization through FTIR, DSC, and TGA were undertaken. The Fourier transform infrared spectra of loxoprofen sodium show characteristic peaks as shown in Supplementary Figure S4D, Ethocel 10P and Methocel K100M spectra showed peaks (see Supplementary Figures S4C, D. Drug excipients spectra showed peaks as shown in Table 5 and Supplementary Figure S5. The Loxoprofen sodium DSC plot showed an endothermic peak (see Figure 2A) Moreover, The TGA plots revealed that loxoprofen sodium losses its mass in two steps (Figure 2B).

**TABLE 5:** Summary of the key findings (characteristic peaks) of FTIR of loxoprofen sodium with respect to methocel K100M, ethocel 10P, sodium bicarbonate and formulation blend (1:1:1:1; incubated for 30 days at 40°C and 75 RH

		Wavelength peak assignments	Hydrogen bonded OH stretching	Asymmetric stretching	Aromatic combination Bands	C=C stretching	OH Bending		H-C-H bending
		Wavelength (Unit)	3,570–3,200 cm^-1^	2,935–2,915 cm^-1^	2000–1,600 cm^-1^	1,680–1,620 cm^-1^	1,410–1,310 cm^-1^		1,190–1,130 cm^-1^
Drug	Loxoprofen Sodium	Characteristic peaks	3,660	2,930	1740	1,680	1,410	1,390	1,180
Excipients	Methocel K100M	Characteristic peaks	3,480	2,950	NIL	1,654	1,410	-	1,180
	Ethocel 10P	Characteristic peaks	NIL	NIL	NIL	(C-H)	1,410	-	1,190
						1,654			
	NaHCO_3_	Characteristic peaks	3,500	NIL	NIL	1,650	1,510	1,330	-
Physical mixture of each components (1:1:1:1) (after 30 days incubation at 40°C/75% ± 5% RH)		Peak shift/appearance/disappearance	3,390	2,940	1760	1,670	1,430	1,310	1,180
		Peak remarks	Shifted	Stretched	Stretched	Stretched	Bending	Bending	Intact
		Interaction type	Only physical interaction	Only physical interaction	Only physical interaction	Only physical interaction	Only physical interaction	Only physical interaction	No Interaction

### Formulation development

An I-optimal mixture design yielded a total of 16 formulations with 40% loxoprofen sodium and 1% magnesium stearate as a processing aid. Remainder of the 59% formulation comprised of varied levels of methocel K100M (34%–45%), ethocel 10P (0%–10%), and NaHCO_3_ (5%–15%) (see Table 3). The experimental responses to the designed formulations, such as floating lag time, total floating time, and drug release at 1, 12, and 24 h Were evaluated and are discussed in the proceeding Sections (see Table 2).

#### Floating lag-time (sec) and total floating time (h)

**FIGURE 3:**
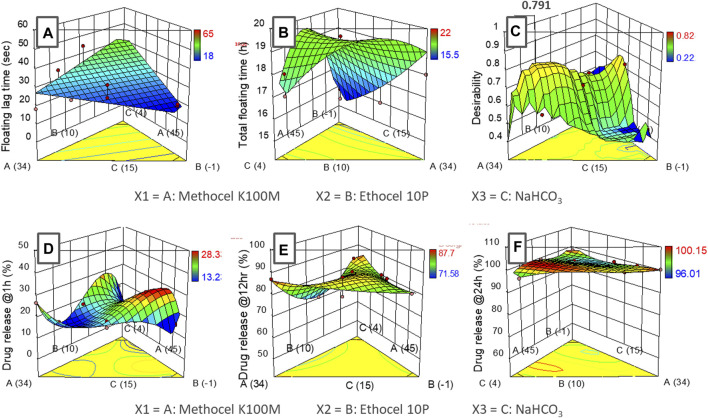
Response surface graphs (I-optimal mixture design) of input variables (methocel K100M, ethocel 10P and NaHCO_3_) with responses **(A)** floating lag-time, **(B)** total floating time, **(C)** desirability **(D)** drug release at 1 h, **(E)** drug release at 12 h and **(F)** drug release at 24 h.

The assessment of the time lapsed till the formulation starts floating at the surface of the media (floating lag-time; sec) (see Table 6; Figure 3A) and In the case of the total floating (h) time (see Table 6 and Figure 3B effect among the three variables were evaluated.

**TABLE 6:** Physicochemical and pharmaceutical evaluation of different loxoprofen sodium gastroretentive floating mini-tablet.

Formulation code	Weight variation (mg)	Friability (%)	Hardness (kg/cm^2^)	Thickness (mm)	Diameter (mm)	Content uniformity (%)	Floating lag-time (s)	Total floating time (h)
F1	15.62 ± 0.70	0.79 ± 0.11	1.37 ± 0.07	2.19 ± 0.029	3.06 ± 0.015	91.29 ± 3.65	19 ± 1.51	16 ± 0.04
F2	15.4 ± 1.03	0.61 ± 0.07	1.27 ± 0.02	2.15 ± 0.024	3.07 ± 0.02	96.7 ± 2.05	35 ± 1.52	22 ± 0.07
F3	15.15 ± 0.82	0.71 ± 0.11	1.39 ± 0.09	2.17 ± 0.029	3.06 ± 0.015	99.12 ± 1.73	21 ± 1.52	21.5 ± 0.15
F4	14.35 ± 0.93	0.87 ± 0.13	1.47 ± 0.9	2.12 ± 0.026	3.05 ± 0.012	95.84 ± 1.92	45 ± 1.52	18 ± 0.12
F5	14.82 ± 1.13	0.80 ± 0.1	1.43 ± 0.11	2.17 ± 0.025	3.09 ± 0.014	91.6 ± 5.19	31 ± 1.52	16 ± 0.18
F6	14.82 ± 0.82	0.86 ± 0.11	1.57 ± 0.08	2.09 ± 0.028	3.08 ± 0.017	91.29 ± 3.65	65 ± 1.52	17 ± 0.07
F7	13.92 ± 0.71	0.81 ± 0.09	1.42 ± 0.14	2.16 ± 0.026	3.09 ± 0.015	89.05 ± 2.61	63 ± 1.52	18 ± 0.08
F8	14.65 ± 1.27	0.69 ± 0.05	1.47 ± 0.02	2.11 ± 0.023	3.1 ± 0.015	88.92 ± 3.93	23 ± 1.52	18 ± 0.11
F9	14.82 ± 0.82	0.84 ± 0.13	1.39 ± 0.08	2.16 ± 0.028	3.09 ± 0.015	93.05 ± 2.65	20 ± 1.52	15.5 ± 0.05
F10	14.85 ± 0.54	0.73 ± 0.12	1.45 ± 0.08	2.17 ± 0.029	3.10 ± 0.016	97.64 ± 1.82	23 ± 1.52	17.5 ± 0.15
F11	15.4 ± 0.82	0.72 ± 0.12	1.51 ± 0.01	2.12 ± 0.022	3.07 ± 0.05	97.4 ± 2.49	18 ± 1.52	18 ± 0.20
F12	14.98 ± 1.01	0.82 ± 0.08	1.43 ± 0.08	2.17 ± 0.027	3.09 ± 0.012	92.46 ± 5.4	26 ± 1.52	18.5 ± 0.12
F13	14.8 ± 1.15	0.83 ± 0.12	1.46 ± 0.1	2.09 ± 0.030	3.06 ± 0.015	92.01 ± 1.91	24 ± 1.52	19 ± 0.05
F14	14.45 ± 1.05	0.79 ± 0.11	1.38 ± 0.08	2.15 ± 0.028	3.1 ± 0.015	92.01 ± 1.82	34 ± 1.52	18.4 ± 0.081
F15	15.1 ± 0.71	0.86 ± 0.13	1.39 ± 0.09	2.14 ± 0.027	3.09 ± 0.014	93.04 ± 4.12	22 ± 1.52	19 ± 0.076
F16	15.6 ± 1.01	0.76 ± 0.12	1.44 ± 0.09	2.14 ± 0.020	3.1 ± 0.016	90.6 ± 3.2	24 ± 1.52	17.5 ± 0.15

#### Drug release, kinetics, and mechanisms

The effect of the three variables upon the drug release at different time points (1, 12, 24 h) was also studied. For this purpose, drug release at 1 h was selected to analyze the burst effect phenomena (see Figure 3D, whereas the release at 12 h was assessed to ascertain the continued progressive increment of the drug coming out of the system (Figure 3E), Moreover, the 24 h interval was selected to undertake maximum release of the drug from the system (see Figure 3F respectively.

**FIGURE 4:**
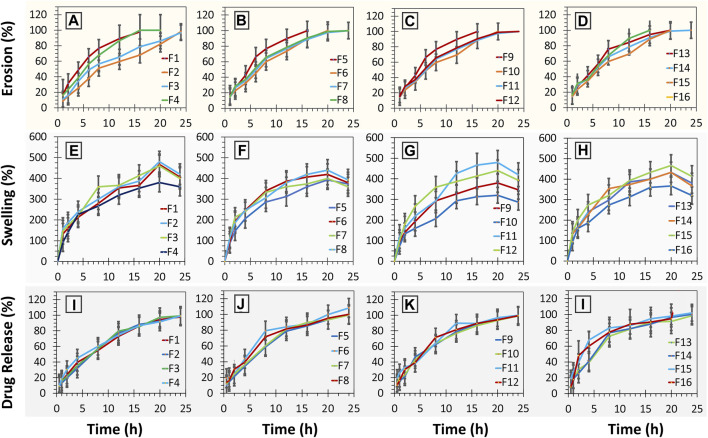
**(A–D)** erosion (%) studies of minitablet formulations (F1-F16) *versus* time; **(E–H)** % swelling studies of minitablet formulations (F1-F16) with respect to time (h); **(I–L)** % drug release from minitablet formulations (F1-F16) with respect to time (h).

In terms of the release pattern, nearly all the formulations were evaluated except F11, which was generally found to release the drugs following the first-order kinetics (see Figures 4I–L; Table 7). Moreover, formulation F2 was also found to depict high conformance to zero-order kinetics (*r* ^*2*^ *= 0.956;* K_O_ = 6.48 h^-1^) as well (see Table 6). Formulations exhibited release by means of diffusion with subsequent swelling and erosion (see Figures 4E–H). Drug release models and mechanism (see Table 7).

**TABLE 7:** Model dependent release kinetics of dissolution profiles of loxoprofen sodium from various gastroprotective mini-tablet (F1-F16) formulations.

	Zero order		First order		Higuchi		Korsmeyer-Peppas			Hixson-Crowell	
Formulation Code	R^2^	K_0_ (*hr* ^*-1*^)	R^2^	K_1_ (*hr* ^*-1*^)	R^2^	K_H_ (*hr* ^*-1/2*^)	R^2^	N	K_kp_ (*hr* ^*-n*^)	R^2^	K_HC_ _*(hr*_^*-1/3*^)
F1	0.77	7.862	0.956	0.114	0.979	19.855	0.994	0.591	16.614	0.929	0.032
F2	0.956	6.848	0.986	0.111	0.926	19.675	0.997	0.745	12.067	0.988	0.033
F3	0.931	7.055	0.977	0.118	0.942	20.405	0.995	0.700	13.717	0.975	0.034
F4	0.735	7.155	0.947	0.133	0.984	21.332	0.988	0.543	19.618	0.900	0.037
F5	0.938	7.057	0.985	0.118	0.94	20.382	0.998	0.710	13.426	0.984	0.034
F6	0.79	8.291	0.949	0.168	0.942	24.448	0.957	0.592	20.422	0.939	0.047
F7	0.909	7.310	0.976	0.127	0.955	21.253	0.996	0.668	15.258	0.971	0.036
F8	0.83	7.822	0.973	0.150	0.963	23.015	0.983	0.605	18.573	0.956	0.042
F9	0.85	7.404	0.98	0.135	0.957	21.723	0.983	0.623	17.065	0.96	0.038
F10	0.802	7.330	0.968	0.135	0.978	21.666	0.991	0.58	18.539	0.938	0.037
F11	0.738	8.063	0.869	0.157	0.958	22.927	0.957	0.547	21.785	0.85	0.043
F12	0.851	7.815	0.982	0.149	0.96	23.102	0.984	0.623	18.029	0.967	0.041
F13	0.849	7.850	0.978	0.151	0.944	23.534	0.982	0.611	18.579	0.961	0.042
F14	0.843	8.027	0.977	0.156	0.934	27.854	0.971	0.627	18.342	0.967	0.044
F15	0.359	9.091	0.952	0.262	0.934	27.854	0.948	0.435	31.511	0.896	0.075
F16	0.601	8.867	0.957	0.227	0.926	26.747	0.927	0.506	26.418	0.912	0.063

#### Morphological and pharmaceutical evaluation

Macroscopically, the prepared tablets were of white to off-white color with no cracks (see Figures 6A–E. Similarly, scanning electron microscopy (SEM) (see Figures 6A–D). Besides morphology, the average weight of mini-tablet ranges from 13.92 ± 0.71 to 15.62 ± 0.70 mg (see Table 6). The average thickness and diameter of mini-tablets range between 2.09 ± 0.030 to 2.19 ± 0.029 and 3.05 ± 0.012–3.1 ± 0.015, respectively (see Table 7). In terms of physical strength, the friability values of the mini-tablets (Table 6). In terms of uniformity of dosage per tablet is concerned, the content was determined to be in the range of 88.92 ± 3.93 to 101.59 ± 2.05 (see Table 6).

#### Formulation optimization, Heckel analysis, and stability studies

The designed and developed loxoprofen sodium mini-tablet formulations were optimized by numerical criteria whereby minimum floating lag-time (sec), maximum total floating time (h), drug release at 1 h (set as “minimum”), 12 h (set as “in-range”) and 24 h (set as “maximum”) were used in the optimization purpose (see Table 3), respectively.

The optimized formulation blend (F2) was subjected to Heckle analysis to assess the optimum compression pressure for the commercial manufacturing of the tablets. The results indicated that the compression profile consisted of two regions in the compression range (see Figure 7).

The optimized formulation was then subjected to accelerated stability testing (40°C/75% ± 5% RH) and its shelf life was calculated using statistical software (Minitab, version 20) (see Supplementary Table S6 for the results data).

### In-silico PBPK modeling and simulation

The optimized formulation (F2) was subjected to *in silico* “physiologic based pharmacokinetics” (PBPK) modeling and simulation. Firstly, for this purpose, the pharmacokinetic data of loxoprofen sodium (immediate-release tablet; 60 mg) formulation from 24 healthy male Korean volunteers was obtained from the study by Kang et al. (2011) (extracted data is given in Supplementary Table S4) and was further processed in the PKPlus™ module of GastroPlus™ (version 9.8; Simulations Plus Inc. Lancaster, CA, United States) (see Table 4). The *in-vivo* simulation was performed in (ACAT) “Advanced Compartmental and Transit” model in GastroPlus™ software and the drug release data of the optimized formulation (F2 drug release data at pH 1.2) was used for that purpose. The *in-vivo* profile (Kang et al. (2011)) of the immediate release tablet 60 mg loxoprofen sodium (see Figure 8) was compared with the simulated profile of the optimized formulation (F2, 120 mg loxoprofen Na; 20 minitablets encapsulated in size “00” capsules, see Figures 6E, F for the picture of capsules and tablets). The calculated values of fold error (FE) and error percentage (%PE) of the simulated data along with the values of various pharmacokinetic parameters (Cmax, T_max_, AUC_t_, AUCinf) are also illustrated in Supplementary Table S5.

## Discussion

### SeDeM based characterization

SeDeM based characterization of loxoprofen sodium (see Figure 1 and Supplementary Tables S1, S2), showed that overall, the drug material was found suitable for direct compression with an ICG value of 5.76, well above the minimum criteria of 5 (see Figure 1 and Supplementary Tables S1, S2) (Suñé-Negre et al., 2008). The SeDeM based investigation for the functional polymers (methocel K15M, K100M and ethocel 10-P) also revealed poor angle of repose. However, this deficiency could be overcome with the use of processing aid (glidant; magnesium stearate) in the formulation blends (Aguilar-Díaz et al., 2009). Besides the angle of repose, the polymers were found with good compressibility and overall satisfactory flowability with average values of the incidence parameters near (dimension), and in most cases, above (compressibility, flowability, etc., lubricity, dosage) the minimum criteria value of 5. The “Good Compressibility Index” (ICG) values were calculated above 5 as well, showing high suitability of all the polymers for the direct compression (see Figure 1 and Supplementary Table S2). The minimum percentages of the excipients for the correction of the API were calculated as, Methocel K15M, 28.46%; K100M, 33.3%, and Ethocel 10P as ∼15%) (see Table 2). Since sodium bicarbonate was to be used in minimal quantities (5%–15%) as shown in (Table 2) as a functional excipient (effervescent agent), its only deficiency in respect of the “compressibility incidence”profile did not matter much in the overall quality expectations of the formulation blends.

### Fourier transform infrared spectroscopy (FTIR)

The Fourier transform infrared spectra of loxoprofen sodium show characteristic peaks such as O-H broad peak at around 3,360 cm^-1^ carboxylic group peak at 1732 cm^-1^. Moreover, asymmetric C-H bond peaks (CH_3_ and CH_2_) attributed due to stretching of aromatic rings were present at 1,646 cm^-1^, 1,612 cm^-1^, 1,440 cm^-1^, 1,402 cm^-1^1391 cm^-1^, 1292 cm^-1^, 1,148 cm^-1^, 723 cm^-1^ and 670 cm^-1^. They are resultant due to the stretching of methylene–CH_2_ bending scissoring as shown in Supplementary Figure S4D (Khalid et al., 2018a). Ethocel 10P spectra showed peaks at 890 cm^-1^, 920 cm^-1^ attributed to the N-H and O-H bending, respectively. The peaks at 1,090 cm^-1^, 1,310 cm^-1^ 1732 cm^-1^ and 2,970 cm^-1^ and 3,359 cm^-1^ corresponding to C-O, CH_2_ and CH_3_ stretching, respectively, were also present [see Supplementary Figure S4C (Pineda and Hechenleitner, 2004]. Methocel K100M spectra showed peaks at 3,361 cm^-1^, 2,971 cm^-1^, 2,923 cm^-1^ 2,359 cm^-1^ 2,341 cm^-1,^ 1733 cm^-1^, 1,410 cm^-1^, 1,180 cm^-1^ and 980 cm^-1^ attributed to stretching of C-H, O-H and C-C, respectively. The spectra of the drug–excipients mixture showed a peak at 3,360 cm^-1^ along with small and narrow peaks at 1740 cm^-1^.1,660 cm^-1^.1612 cm^-1^.1,440 cm^-1^.1,440 cm^-1^, 1,361 cm^-1^ and 1,292 cm^-1^ (see Supplementary Figure S4B). The peaks in the physical mixture of drug and excipients show slight shifting due to the physical interaction between drug and excipients. However, no peaks were observed to appear or disappear correspondingly to any chemical interaction of the ingredients in the mixture. This showed that no potential drug–excipients interactions were observed. Loxoprofen sodium peaks were found that excipients did not induce any incompatibility except for a few physical interactions phenomena (Supplementary Figure S5 and Table 5) (Zaman et al., 2013b).

### Differential scanning calorimetry (DSC) and thermogravimetric analysis (TGA)

Loxoprofen sodium DSC plot showed a peak (see Figure 2A) as an endothermic peak at ∼89°C, 204°C, and at 435°C, corresponding to the loss of moisture, melting, and thermal degradation of the drug, respectively. In the physical mixture of drug and excipients, the characteristic peak of the drug for its melting point was found intact respective to the proportion of the drug in the mixture suggesting no chemical interaction of the constituents. Similarly, the loss of moisture was correspondingly similar in the physical mixture of drug and excipients as was expected for the loss in the drug and excipients individually in the broad range of ∼70°C–100°C (see Figure 2A). Moreover, the range at which drug degradation happens was relatively unchanged due to the overall masking effect since polymers in the formulation would exhibit an exothermic reaction during their degradation in the same temperature region (Pereira et al., 2013; Zaman et al., 2015a). Moreover, The TGA plots revealed that loxoprofen sodium losses its mass in two steps (see Figure 2). Firstly, approximately 10% mass is lost between 85°C and 105°C corresponding to the loss of the moisture embedded in the particulate matter and subsequently in the next phase loss of around 8% and ∼52% happens in two steps consecutively between 290°C and 380°C. and then 390°C–490°C, respectively. This two steps loss is attributed to the thermal degradation of the drug subsequent to the melting (Khalid et al., 2018b; Farooq et al., 2019). TGA results of the physical mixture of drug and excipients revealed a similar profile as of the individual components after 30 days of incubation under stress conditions (Ali et al., 2017; Khalid et al., 2018c).

### Formulation development

The experimental responses to the designed formulations, such as floating lag time, total floating time, and drug release at 1, 12, and 24 h (see Table 2), were evaluated and are discussed in the proceeding sections.

#### Floating lag-time (sec) and total floating time (h)

The assessment of the time lapsed till the formulation starts floating at the surface of the media (floating lag-time; sec) (see Table 6; Figure 3A) which, suggested a linear relationship (*p < 0.05*) for the three formulation variables. Among the three variables, NaHCO_3_ and ethocel 10P were found to have a significant effect on the response. As expected, the lag time (see Table 6 and Figure 3B) was inversely proportional to the amount of effervescent agent (NaHCO_3_; ANOVA: *p ∼ 0.05*) while the proportion of water insoluble polymer (ethocel 10P) was found to influence the lag time (ANOVA: *p < 0.1*) mildly in a proportionate way (Wasilewska and Winnicka, 2019). This could be because a higher proportion of the polymer would retard the penetration of water inside the core of the tablet, thus rendering lesser chances for effervescent reaction with the NaHCO_3_ crystals present in the core.

In the case of the total floating (h) time (figure b), a quadratic model was found to represent the relationship of the variables with the response with a low statistical significance (ANOVA: *p < 0.1*). However, ethocel 10P and NaHCO_3_ proportions as a combined factor was found to have a relatively higher significance (ANOVA: *p < 0.05*). The higher proportion of ethocel 10 P was found to contribute to significantly higher floating times (>18 h) (see Figure 3B. This fact could be attributed to the entrapment of CO_2_ gas bubbles (produced after effervescent reaction) for a longer period of time inside the core tablets with higher levels of water insoluble polymers and thus decreasing the density of the core (Gutiérrez-Sánchez et al., 2008). The proportion of methocel K100M used in the experiments (34%–45%) was found to have an insignificant effect on the responses.

#### Drug release, kinetics, and mechanisms

The data fitting of the drug release at 1 h followed cubic model representation (ANOVA; *p < 0.01*). The drug release was found to be more retarded with higher percentages of water-insoluble polymer, ethocel 10P, in combination with methocel K100M. It was also observed that increasing the content of NaHCO_3_ led to the burst release phenomena with up to ∼26% release in the first hour (see Figure 3D; Table 2). This could be due to the generation of water channels in the matrix after the dissolution of NaHCO_3_ particles (Nagarwal et al., 2010). The data of drug release at 12 h exhibited a special cubic model fitting with high significance for effect of the variables upon the response (ANOVA: *p < 0.05*). The drug release varied between ∼71–87% with the highest release observed around the center region of the variable’s proportions. The lower release was only observed at higher proportion of ethocel 10 P (see Table 2 and Figure 3E (Wasilewska and Winnicka, 2019). Furthermore, the release at 24 h did not demonstrate any significance of relationship of variables with responses with lower order model fitting (quadratic and cubic) and was found in the range of 96%–100% (see Figure 3F.

In terms of the release pattern, nearly all the formulations were evaluated except F11, were generally found to release the drugs following the first-order kinetics (see Figures 4I–L; Table 7). Moreover, formulation F2 was also found to depict high conformance to zero-order kinetics (*r* ^*2*^ *= 0.956;* K_O_ = 6.48 h^-1^) as well. In terms of mechanistic modeling of the drug release, the Korsmeyer-Peppas suggested that most of the formulations released the drug largely by means of Fickian-diffusion as depicted by the “*n*” values ranging from ∼0.5 to ∼0.62. However, formulations F2 (*n* = 0.745), F3 (*n* = 0.70), F5 (*n* = 0.710) and F7 (*n* = 0.668) exhibited the release by means of diffusion as well as swelling of the matrix with subsequent erosion of the diffusion-front layer (Non-Fickian, anomalous transport) (Siepmann and Goepferich, 2001; Siepmann and Siepmann, 2008). This phenomenon can also be visually confirmed by the swelling studies, whereby the formulations were observed to swell to a cumulative ∼490 percent of the original size (see Figures 4E–H. The formulations with a higher proportion of methocel K100M with a consequently lower proportion of ethocel 10P tend to swell at a faster pace as seen in the cases of formulations F5 and F15 (see Figures 4F, H).

Overall, the swelling phenomenon occurred with simultaneous shrinking due to erosion of the diffusion-front of the matrices. The combined effect would result in an overall reduction in the size of the tablets as observed for almost all the formulations ranging from ∼16 to 20 h. As expected, the formulations (F2 and F6) with a larger proportion of ethocel 10P demonstrated a relatively slow erosion process (see Figures 4A, B). This is most likely due to the formation of water insoluble front that does not let the polymer become hydrated enough for erosion. Moreover, certain formulations (F10 and F16) with a higher proportion of NaHCO_3_ were found to exert relatively faster erosion phenomena at a later time stage (>10 h) even in the presence of ethocel 10 P (see Figures 4C, D). This is possibly owed to the dissolution of NaHCO_3_ particles after a certain time and resulting water imbibing in the core with consequent erosion. On the contrary, the formulations with no or low proportions of ethocel 10P (F1, F5, and F9) along with higher proportions of NaHCO_3_ did show faster erosion processes even early as ∼4 h (50% erosion for F1) (see Figures 4A–D).

**FIGURE 5:**
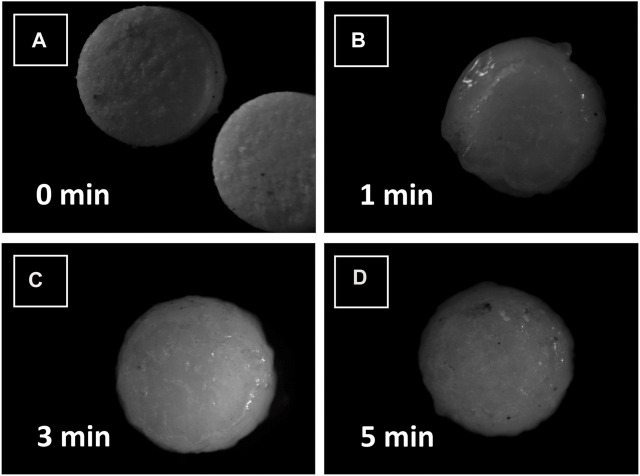
Stereomicrographs of loxoprofen sodium gastroretentive minitablet formulations after contacting with water **(A)** at 0 min, **(B)** after 1 min, **(C)** after 3 min and **(D)** after 5 min of contact with water.

Furthermore, since the mini-tablet formulations have a larger surface area and lesser aspect ratio (diameter vs. thickness) as compared to large size conventional formulations, they tend to exhibit a shape close to a sphere upon swelling (see Figures 5A–D) (Siepmann and Siepmann, 2008). This, in return, indicates the release of drugs to follow Hixon-Crowell (shrinkage of size with cube-root function) kinetics, which was observed for almost all the formulations (see Table 7).

#### Morphological and pharmaceutical evaluation

**FIGURE 6:**
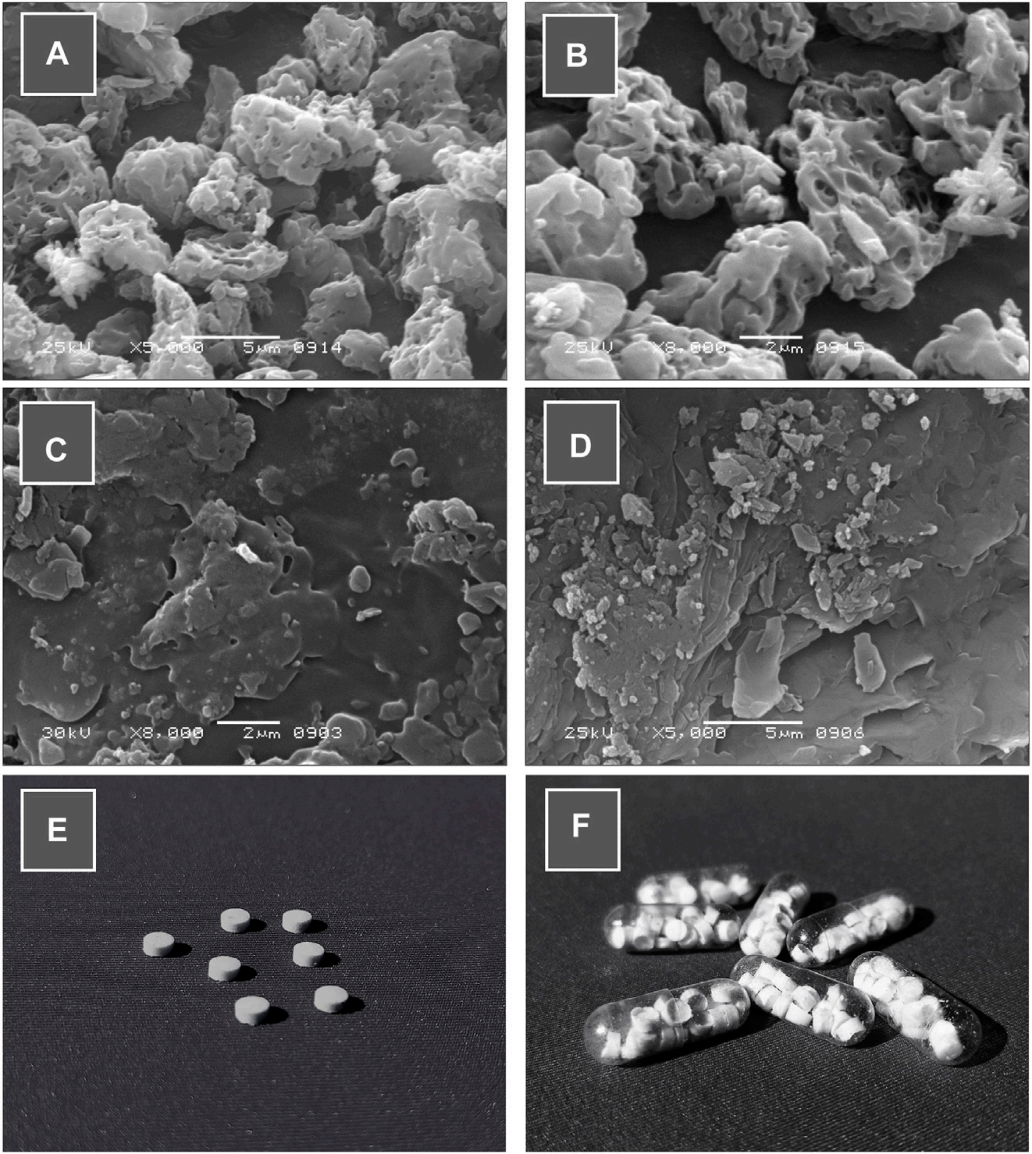
**(A ,B)** Scanning electron microscopic (SEM) images of physical mixture of formulation blend, **(C ,D)** SEM images of surface morphology of the compressed minitablets, **(E)** Photographic image of loxoprofen Na gastroretentive minitablets and **(F)** capsules (size “00”) filled with 20 minitablets (120 mg loxoprofen Na).

Macroscopically, the prepared tablets were of white to off-white color with no cracks (see Figures 6A–E. Upon examining the formulation blend by scanning electron microscopy (SEM) (see Figures 6A–D it was revealed that the particulate matter of the blend was well distributed and well mixed in the size range of 10–30 µm. However, upon compression, these particulate materials appeared to be somewhat fused up with each other, possibly due to compressional pressure, and exhibited a flaky or fused platy structure that formed the tablet surface layers. (see Figures 6A–D). Besides morphology, the average weight of mini-tablet ranges from 13.92 ± 0.71 to 15.62 ± 0.70 mg (see Table 6). All tablets passed the weight variation test as it was found to be within ±10% as per the pharmacopoeial requirements. The average thickness and diameter of mini-tablets range between 2.09 ± 0.030 to 2.19 ± 0.029 and 3.05 ± 0.012–3.1 ± 0.015, respectively (see Table 6).

In terms of physical strength, the friability of the mini-tablets was determined to be acceptable as it was lesser than 1% (range 0.61–0.86) for all the formulations (see Table 6). Moreover, the hardness was found to be in the range of 1.27–1.57 kg/cm^2^ (see Table 6) (Louis et al., 2020c). In terms of uniformity of dosage per tablet is concerned, the content was determined to be in the range of 88.92 ± 3.93 to 101.59 ± 2.05 and was found to be within the pharmacopoeial limit (El-Zahaby et al., 2014). Table 6 shows all the values of physicochemical properties.

The optimization procedure revealed a formulation with 36% methocel K100M, ∼10% ethocel 10P and ∼12.5% NaHCO_3_ with a desirability value of ∼0.79 for the criteria (see Figure 3C). The desired output value for the optimized formulation as per the responses was found to exhibit the floating lag time of ∼30 s, total floating time of ∼20 h, and drug release at 1, 12, and 24 h as ∼13, ∼77, and ∼99%, respectively. The formulation F2 with the closest formulation proportions of the excipients was therefore chosen as the optimized formulation.

**FIGURE7:**
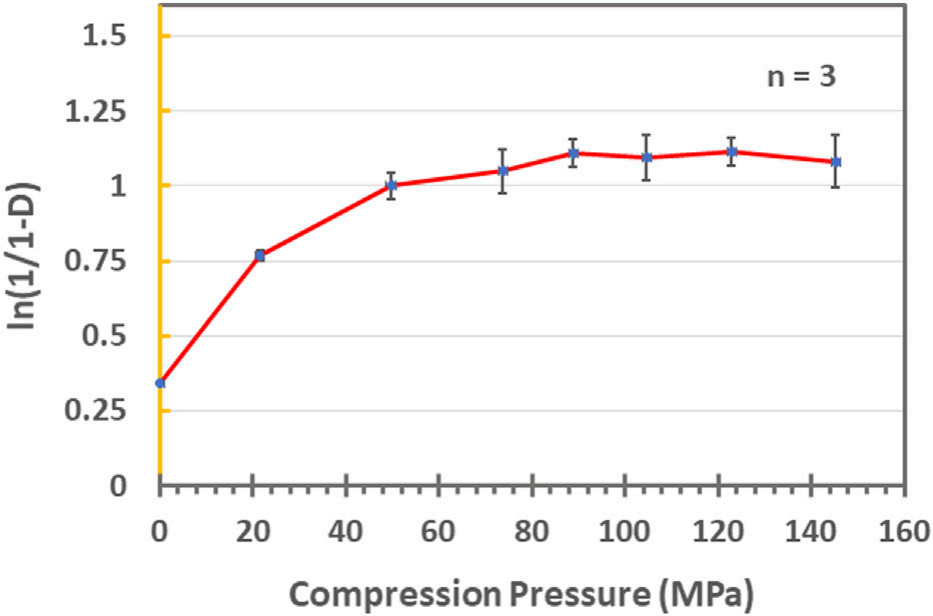
Heckle plot of the optimized formulation-blend (F2) at various pressure values (0–160 MPa).

The optimized formulation blend (F2) was subjected to Heckle analysis to assess the optimum compression pressure for the commercial manufacturing of the tablets (see Figure 7). The results indicated that the compression profile consisted of two regions in the compression range. The first region characterized by particle rearrangement (0–20 MPa) was followed by deformation (∼20–100 MPa) with a smooth transition into the plateau phase characterized by the work hardening of the particles. The yield pressure was calculated to be around 157 MPa (see Figure 7).

The optimized formulation was then subjected to accelerated stability testing (40°C/75% ± 5% RH) for 6 months after encapsulating 20 tablets of loxoprofen sodium (120 mg loxoprofen sodium; 20 tablets in size “00” capsule) (see Figures 6E–F). The results revealed the formulation was stable in terms of its physical attributes such as hardness and friability as well as functional attributes such as floating-lag time, total floating time, drug release in 24 h and content uniformity (see Supplementary Table S5). The shelf-life was calculated to be ∼25 months which can be extended further with proper packaging of the tablets (Zaman et al., 2015b).

### *In-vivo* PBPK based modeling and simulation

**FIGURE 8:**
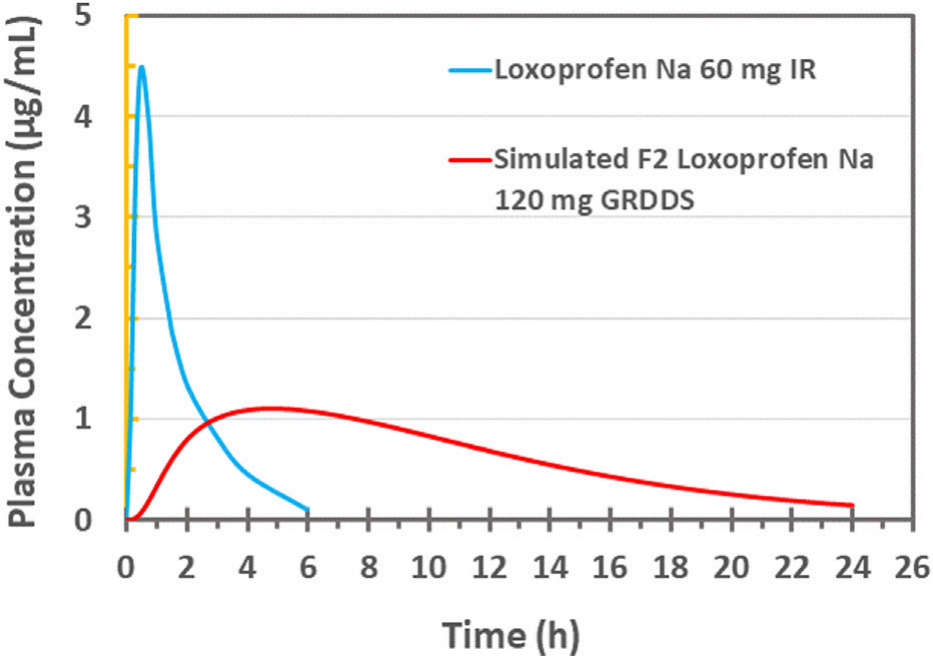
Plasma drug concentration vs. time plot of 60 mg IR loxoprofen sodium administered to healthy Korean volunteers (Kang et al. (2011)) and PBPK based *in silico* modeling and simulation based plasma drug concentration of 120 mg loxoprofen sodium GRDDS minitablets.

Various pharmacokinetics parameters such as *C*_max_ (4.866 ug/mL), *T*_max_ (0.4812 h), *AUC*_*0-t*_ (7.364 ug/mL×h), *AUC*_*T-inf*_ (7.498 ug/mL×h), *C*_*L*_ (7.342 L/h), *V*_*D*_ (0.0381 L/kg), *K*_*12*_ (0.3013 h^-1^) and *K*_*21*_ (0.02116 h^-1^) for the drug were estimated after fitting of the PK data for the two compartment model (Kang et al., 2011) (Figure 8). This data, along with other physicochemical and physiologic data of the drug, was incorporated in the “Absorption and Continuous Transit” (ACAT) model of the Gastroplus™ (see Table 4). The *in-vitro* drug release data (pH 1.2) of the optimized formulation F2 (20 tablets in size “00” capsules; 120 mg loxoprofen sodium; Figures 6E, F) was then subjected to simulation (see Figure 8). The results revealed a *Cmax* of 1.0958 ug/mL and *Tmax* 4.8 h for the once-daily formulation (F2). Moreover, the plasma concentration was maintained for around 24 h with *AUC0-t* 14.448 ug/mL×h and *AUC0-Inf* 15.459 ug/mL×h. The relative bioavailability of the optimized formulation was calculated to be 97.0%. Since the developed controlled release formulation has a double quantity of API as compared to IR 60 mg tablet, so the total amount of drug reaching the systemic circulation would be expected twice which is verified by the values of AUC. Therefore, the calculations of FE and %PE were made with twice the values of AUC and the results were found satisfactory (FE = 1.03 and 0.98; %PE = 3.09% and 1.90% for AUC0 to inf and AUC0 to t, respectively) (See Supplementary Table S5). Thus, the optimized floating multi-unit gastroretentive DDS (20 tablets in a capsule) is sufficient for loxoprofen sodium as a once-daily regimen (Yamakawa et al., 2010b; Mizukami et al., 2013).

## Conclusion

SeDeM assessment revealed that the mixture of loxoprofen sodium, methocel K100M, ethocel 10P, and sodium bicarbonate are suitable for the direct compression technique. Upon drug-excipient compatibility studies, the mixture was found to be physically and chemically stable. The response surface methodology (RSM) revealed that higher proportions of ethocel 10P helped increase the total floating time and extend the drug release for up to 24 h. NaHCO_3_ proportion was also noted to affect the floating-lag time and over all diffusion/swelling mechanism of the drug release. The optimum formulation containing 20 units of loxoprofen sodium in a capsule was determined to give a sufficient half-life (∼25 months) without significant packaging. Upon PBPK-based modeling and simulation, the plasma profile is expected to be within the sufficiency range/levels to ensure loxoprofen sodium administration for once daily.

## Data Availability

The raw data supporting the conclusion of this article will be made available by the authors, without undue reservation.
