# Supplementary material for: SeDeM expert system with I-optimal mixture design for oral multiparticulate drug delivery: An encapsulated floating minitablets of loxoprofen Na and its in silico physiologically based pharmacokinetic modeling
Source: Front Pharmacol. 2023 Mar 3;14:1066018. doi: 10.3389/fphar.2023.1066018 (PMC10022826; doi:10.3389/fphar.2023.1066018)
Supplement: Supplementary file 1 [file DataSheet1.docx]

**Supplementary data file**

**Methodology**

Determination of SeDeM parameters

Twelve basic Sedem parameters were studied with method as descried in European pharmacopoeia.as shown in table 1.

Method described in Section 2.9.15 of European Pharmacopoeia was used to determine bulk density (D_a_),

Bulk density (Da**_)_**

Bulk density (Da) was calculated using equation (1) as mentioned below.

$$bulk density (Da)=\frac{mass \left( m \right)}{bulk volume \left( Vb \right)}\ldots\ldots\ldots\ldots\ldots\ldots\ldots\ldots\ldots\ldots\ldots\ldots\ldots\ldots\ldots\ldots\ldots(1).$$

The total volume in bulk density measurements included particle volume, inter-particle void volume and internal pore volume.

Tapped density (D_c_)

Tapped density was calculated by applying controlled packing force to the sample and includes both pore volume and intestinal volume. Tapped density was determined using method described in Section 2.9.15 of European Pharmacopoeia. D_C_ was determined using graduated cylinder, tapped volume was the value obtained after 2500 strokes. Tapped was determined as shown in equation (2).

$$tapped density (Dc)=\frac{mass\left( m \right)}{tapped \left( Vtap \right)}\ldots\ldots\ldots\ldots\ldots\ldots\ldots\ldots\ldots\ldots\ldots\ldots\ldots\ldots\ldots.(2$$

Inter-particle porosity (Ie)

The drug powder inter-particle porosity (Ie) was calculated by the following equation (3) as mentioned below.

$$inter-particle porosity\left( Ie \right)=\frac{Dc-Da}{Dc\times Da} \ldots\ldots\ldots\ldots\ldots\ldots\ldots\ldots\ldots\ldots\ldots\ldots\ldots\ldots\ldots\ldots\ldots\ldots\ldots\ldots\left( 3 \right)$$

Carr’s index (IC %)

(IC %) was calculated from experimental data using values bulk density (Da), tapped density (Dc) using the equation (4) as mentioned below.

$$Cars indexIC \%=\frac{\left( Dc-Da \right)}{Dc}\times100\ldots\ldots\ldots\ldots\ldots\ldots\ldots\ldots\ldots\ldots\ldots\ldots\ldots\ldots\ldots\ldots\ldots\ldots\ldots\ldots\ldots\ldots\ldots\ldots.\left( 4 \right)$$

Cohesion index (Icd)

The cohesion index was determined by directly compressing the powder under study using an eccentric press. The hardness (N) of the obtained tablets was determined and the mean hardness was calculated. Accurate weight of tablets for cohesion index study can be determined based on bulk density of powders i.e bulk density > 0.70 g/cm^3^ tablet weight is 1.225mg ±0.075, bulk density < 0.40 g/ cm^3^tablet weight is 0.7200 ±0.020 and when bulk density ranges between 0.40 g/cm^3^-0.70 g/cm^3^ 1000mg ±0.050. Compression of tablets should be carried out at the maximum compression force.

Angle of repose (α).

(100 gm) of Powder material was evaluated by passing through a funnel with the dimensions i.e height approximately of 9.5 cm with upper spout's and lower end diameters as approximately 7.2 cm and 1.8 cm respectively. The funnel was kept as high as 20 cm from the base of experimental bench. The material to be studied was filled in the funnel at the top and then the plug is removed to allow the powder to flow on a base to form a cone. The base of the cone ‘r’ was measured four times using a sliding Vernier's caliper and mean radius value was then incorporated in the following formula along with the cone height ‘h' to calculate the angle with the equation (5). As mentioned below.

$\tan\alpha=\frac{h}{r}$ ………………………

Flowability (t″).

Time of flow /Flowability of subjected powder samples was measured in tenth of a second using 100 g of samples. Experiment was performed in triplicate, time of the flow was measured using a digital stopwatch. Mean value of three reading was calculated as method descried in section 2.9.16-2 of Eur. pharmacopoeia.

Loss on drying (% HR)

Accurately weighed 10 gm powder samples were kept for drying in hot air oven adjusted at 105 °C for 2 h until no further weight loss of powder was found (constant weight achieved). Each sample was repeated three time as descried earlier in section 2.2.32. European pharmacopoeia 2011

Hygroscopicity (% H)

Completely dried and accurately weighed Petri dish was taken, 10gm powder sample was poured and kept in stability chamber set at 75% relative humidity with 22.5 °C temperature for period of 24 h. After the aforementioned time sample was taken out from chamber and increase in sample mas was calculated, experiment performed in triplicate. Percentage (%) mass gain was recorded.

Particle size under 50μm size (% Pf)

As described in section .9.12 of European pharmacopoeia 100gm powder sample was taken, particles percentage less than of 50 μm size were determined by using 0.05 mm sieve mounted on a gyratory shaker for about 10 min. mean value of three readings was determined

Homogeneity index (Iθ)

As described in General method in section 2.9.12 European pharmacopoeia. Homogeneity index (Iθ) was determined using a series of sieves (0.355 mm, 0.212 mm, 0.100 mm and 0.05 mm) fitted on top of a gyratory shaker for about 10 mins. 100gm of powder sample was subjected to analysis. Three values of each subjected sample were taken for calculation of index, which is calculated using mathematical formula given below.

$I\theta=\frac{fm}{\begin{aligned} 100+\left( dm-dm-1 \right)fm-1+\left( dm+1-dm \right)fm+1+\left( dm-dm-2 \right)fm-2+\left( dm+2- \right)fm+2+\ldots.+\left( dm-dm-n \right)3 fm-n+\left( dm+n3-dm \right)fm+n3 \\ \\ \end{aligned}} \ldots\ldots\ldots(6)$i

Iθ= relative homogeneity index , ‘fm’ and ‘dm’ is the percent proportion of particles in major size range and their diameter respectively, ‘Fm-1’ ‘and dm-1’ percentage of particles of the size fraction and diameter below the majority size range ‘Fm+1’ and ‘Fm+1’ percentage of particles and diameter of the size.

Based on the physical characteristics of powders (API+ excipients) above mentioned 12parameters were categorized into five factors and their limit values and acceptable ranges are as shown in table1.

Criteria for acceptability of suitability for direct compression

Certain indexes are calculated based on SeDeM diagram through which we determined either the

The product is suitable for direct compression or not. These indexes include parameter index (IP), parameter profile index (IPP), and good compressibility index (IGC). Formulas of all these indexes are as shown below.

*Parameter index* (IP) =$\frac{No.p\geq5}{No.pt}\ldots\ldots\ldots\ldots\ldots\ldots\ldots\ldots\ldots\ldots\ldots\ldots\ldots\ldots\ldots.$ (7)

No. of p ≥ 5: parameters whose values are greater than or equal to 5.

No. pt.: total number of parameters studied

*IP≥0.5 considered acceptable.

Parameter profile index (IPP):

**Supplementary tables**

**Table S1**. Incidence parametric values of loxoprofen sodium, excipients and formulations mixture blend(F1-16)

| **Excipients** | **Bulk density**  **(Da)** | **Tapped density**  **(Dc)** | **Inter**  **particle porosity(Ie)** | **Carrs index**  **(IC)** | **Cohesion index(Icd)** | **Hausner ratio(IH)** | **Angle of repose(α)** | **Powder flow(t")** | **Loss on drying(%HR)** | **Hygroscopicity**  **(%H)** | **Particle <50(%PF)** | **Homogeneity**  **Index**  **(Iθ)** |  |
| --- | --- | --- | --- | --- | --- | --- | --- | --- | --- | --- | --- | --- | --- |
| HPMCK00M | 0.38 | 0.53 | 0.69 | 26.84 | 198.08 | 1.36 | 41.53 | 0.00 | 4.8 | 6.97 | 36.82 | 0.019 |  |
| EC10 PREMIUM | 0.40 | 0.76 | 1.18 | 47.58 | 186.31 | 1.90 | 38.18 | 0.00 | 3.9 | 4.3 | 16.58 | 0.018 |  |
| HPMC K15M | 0.35 | 0.47 | 0.72 | 25.35 | 174.54 | 1.33 | 45.70 | 5.0 | 3.3 | 4.39 | 35.20 | 0.018 |  |
| Na2CO3 | 0.79 | 0.96 | 0.22 | 17.46 | 0 | 1.21 | 33.93 | 4.00 | 4.9 | 2.93 | 18.35 | 0.019 |  |
| Loxoprofen sodium (API) | 0.40 | 0.55 | 0.68 | 27.51 | 28.33 | 1.37 | 39.88 | 4.00 | 3.8 | 2.91 | 4.74 | 0.016 |  |
| **Formulation blend (F1-F16)** | | | | | | | | | | | | | |
| F1 | 0.44 | 0.60 | 0.61 | 25.47 | 99.38 | 1.33 | 39.3 | 2.18 | 4.37 | 4.69 | 20.9 | 0.018 |  |
| F2 | 0.44 | 0.61 | 0.67 | 27.75 | 102.38 | 1.39 | 39.2 | 2.10 | 4.27 | 4.51 | 19.3 | 0.018 |  |
| F3 | 0.42 | 0.58 | 0.66 | 26.91 | 109.01 | 1.36 | 39.6 | 1.98 | 4.32 | 4.77 | 21.0 | 0.018 |  |
| F4 | 0.43 | 0.60 | 0.68 | 27.89 | 107.11 | 1.39 | 39.4 | 2.00 | 4.28 | 4.61 | 19.8 | 0.018 |  |
| F5 | 0.45 | 0.61 | 0.63 | 26.46 | 98.13 | 1.35 | 39.2 | 2.20 | 4.32 | 4.54 | 19.9 | 0.018 |  |
| F6 | 0.41 | 0.58 | 0.70 | 28.40 | 117.14 | 1.40 | 39.7 | 1.80 | 4.27 | 4.81 | 20.7 | 0.018 |  |
| F7 | 0.41 | 0.58 | 0.70 | 28.40 | 117.14 | 1.40 | 39.7 | 1.80 | 4.27 | 4.81 | 20.7 | 0.018 |  |
| F8 | 0.45 | 0.61 | 0.65 | 26.98 | 97.61 | 1.37 | 39.1 | 2.20 | 4.30 | 4.47 | 19.3 | 0.018 |  |
| F9 | 0.44 | 0.60 | 0.61 | 25.47 | 99.38 | 1.33 | 39.3 | 2.18 | 4.37 | 4.69 | 20.9 | 0.018 |  |
| F10 | 0.42 | 0.58 | 0.66 | 26.91 | 109.01 | 1.36 | 39.6 | 1.98 | 4.32 | 4.77 | 21.0 | 0.018 |  |
| F11 | 0.45 | 0.62 | 0.63 | 27.51 | 97.31 | 1.38 | 39.0 | 2.20 | 4.28 | 4.40 | 18.8 | 0.018 |  |
| F12 | 0.43 | 0.60 | 0.68 | 27.89 | 107.11 | 1.39 | 39.4 | 2.00 | 4.28 | 4.61 | 19.8 | 0.018 |  |
| F13 | 0.43 | 0.60 | 0.65 | 27.06 | 104.09 | 1.37 | 39.3 | 2.07 | 4.31 | 4.63 | 20.2 | 0.018 |  |
| F14 | 0.42 | 0.59 | 0.68 | 27.68 | 110.45 | 1.38 | 39.5 | 1.94 | 4.29 | 4.72 | 20.5 | 0.018 |  |
| F15 | 0.44 | 0.60 | 0.63 | 26.30 | 102.46 | 1.35 | 39.4 | 2.11 | 4.34 | 4.67 | 20.6 | 0.018 |  |
| F16 | 0.45 | 0.61 | 0.63 | 26.46 | 98.13 | 1.35 | 39.2 | 2.20 | 4.32 | 4.54 | 19.9 | 0.018 |  |

**Table S2:** Radius parameters for excipients, loxoprofen sodium and formulation blends (F1-F16)

| Excipients | Da | Dc | Ie | IC | Icd | IH | α | T | %HR | %H | Particle <50 | Homogeneity |
| --- | --- | --- | --- | --- | --- | --- | --- | --- | --- | --- | --- | --- |
| Methocel K100M | 3.89 | 5.32 | 5.75 | 5.37 | 9.90 | 8.16 | 1.69 | 10 | 5.20 | 6.51 | 2.64 | 9.61 |
| Ethocel 10P | 4.03 | 7.69 | 9.83 | 9.52 | 9.32 | 5.46 | 2.36 | 10 | 6.10 | 7.85 | 6.68 | 9.32 |
| NaHCO3 | 7.7 | 9.6 | 2.08 | 3.9 | 0.9 | 8.8 | 2.0 | 5 | 3.6 | 8.5 | 0.0 | 10 |
| Loxoprofen sodium | 4.02 | 5.54 | 5.71 | 5.50 | 1.42 | 8.10 | 2.02 | 8.00 | 6.20 | 8.54 | 9.05 | 8.47 |
| Formulation blend (F1-F16) | | | | | | | | | | | | |
| F1 | 4.40 | 6 | 5.08 | 5.09 | 4.97 | 8.35 | 2.14 | 8.91 | 5.63 | 7.66 | 5.82 | 9 |
| F2 | 4.50 | 6.1 | 5.58 | 5.55 | 5.12 | 8.05 | 2.16 | 8.95 | 5.73 | 7.75 | 6.14 | 9 |
| F3 | 4.20 | 5.8 | 5.50 | 5.38 | 5.45 | 8.20 | 2.08 | 9.01 | 5.68 | 7.62 | 5.8 | 9 |
| F4 | 4.30 | 6 | 5.67 | 5.58 | 5.36 | 8.05 | 2.12 | 9.00 | 5.72 | 7.70 | 6.04 | 9 |
| F5 | 4.50 | 6.1 | 5.25 | 5.29 | 4.91 | 8.25 | 2.16 | 8.90 | 5.68 | 7.73 | 6.02 | 9 |
| F6 | 4.10 | 5.8 | 5.83 | 5.68 | 5.86 | 8.00 | 2.06 | 9.10 | 5.73 | 7.60 | 5.86 | 9 |
| F7 | 4.10 | 5.8 | 5.83 | 5.68 | 5.86 | 8.00 | 2.06 | 9.10 | 5.73 | 7.60 | 5.86 | 9 |
| F8 | 4.50 | 6.1 | 5.42 | 5.40 | 4.88 | 8.15 | 2.18 | 8.90 | 5.7 | 7.77 | 6.14 | 9 |
| F9 | 4.40 | 6 | 5.08 | 5.09 | 4.97 | 8.35 | 2.14 | 8.91 | 5.63 | 7.66 | 5.82 | 9 |
| F10 | 4.20 | 5.8 | 5.50 | 5.38 | 5.45 | 8.20 | 2.08 | 9.01 | 5.68 | 7.62 | 5.8 | 9 |
| F11 | 4.50 | 6.2 | 5.25 | 5.58 | 4.87 | 8.10 | 2.2 | 8.90 | 5.72 | 7.80 | 6.24 | 9 |
| F12 | 4.30 | 6 | 5.67 | 5.58 | 5.36 | 8.05 | 2.12 | 9.00 | 5.72 | 7.70 | 6.04 | 9 |
| F13 | 4.30 | 6 | 5.42 | 5.41 | 5.20 | 8.15 | 2.14 | 8.97 | 5.69 | 7.69 | 5.96 | 9 |
| F14 | 4.20 | 5.9 | 5.67 | 5.54 | 5.52 | 8.10 | 2.1 | 9.03 | 5.71 | 7.64 | 5.9 | 9 |
| F15 | 4.40 | 6 | 5.25 | 5.26 | 5.12 | 8.25 | 2.12 | 8.95 | 5.66 | 7.67 | 5.88 | 9 |
| F16 | 4.50 | 6.1 | 5.25 | 5.29 | 4.91 | 8.25 | 2.16 | 8.90 | 5.68 | 7.73 | 6.02 | 9 |

**TableS3:** Mean incidence values and parameter index for excipients, API (Loxoprofen sodium) and of formulation blends(F1-F16)

| Excipients | Dimension | compressibility | Flow ability | lubricity | dosage | Mean incidence values | IP | IPP | IGC | Acceptability |
| --- | --- | --- | --- | --- | --- | --- | --- | --- | --- | --- |
| HPMCK00M | 4.61 | 7.01 | 6.62 | 5.86 | 6.12 | 6.04 | 0.75 | 6.17 | 5.87 | A |
| EC10 PREMIUM | 5.86 | 9.56 | 5.94 | 6.97 | 8.00 | 7.26 | 0.83 | 7.35 | 6.99 | A |
| HPMC K15M | 4.12 | 6.60 | 5.55 | 7.25 | 6.20 | 5.94 | 0.83 | 5.97 | 5.68 | A |
| Na2CO3 | 8.78 | 1.78 | 6.72 | 6.82 | 8.89 | 6.59 | 0.50 | 6.20 | 5.91 | A |
| Loxoprofen sodium | 4.78 | 4.21 | 6.04 | 7.37 | 8.76 | 6.23 | 0.75 | 6.05 | 5.76 | A |
| **Formulation blend(F1-F16)** | | | | | | | | | | |
| F1 | 5.2 | 5.0 | 6.5 | 6.6 | 7.4 | 6.15 | 0.75 | 6.08 | 5.79 | A |
| F2 | 5.3 | 5.4 | 6.4 | 6.7 | 7.6 | 6.28 | 0.75 | 6.21 | 5.91 | A |
| F3 | 5.0 | 5.4 | 6.4 | 6.6 | 7.4 | 6.18 | 0.83 | 6.14 | 5.84 | A |
| F4 | 5.2 | 5.5 | 6.4 | 6.7 | 7.5 | 6.26 | 0.83 | 6.21 | 5.91 | A |
| F5 | 5.3 | 5.1 | 6.4 | 6.7 | 7.5 | 6.22 | 0.75 | 6.14 | 5.84 | A |
| F6 | 5.0 | 5.8 | 6.4 | 6.7 | 7.4 | 6.24 | 0.83 | 6.21 | 5.91 | A |
| F7 | 5.0 | 5.8 | 6.4 | 6.7 | 7.4 | 6.24 | 0.83 | 6.21 | 5.91 | A |
| F8 | 5.3 | 5.2 | 6.4 | 6.7 | 7.6 | 6.25 | 0.75 | 6.17 | 5.87 | A |
| F9 | 5.2 | 5.0 | 6.5 | 6.6 | 7.4 | 6.15 | 0.75 | 6.09 | 5.79 | A |
| F10 | 5.0 | 5.4 | 6.4 | 6.6 | 7.4 | 6.18 | 0.83 | 6.14 | 5.84 | A |
| F11 | 5.4 | 5.2 | 6.4 | 6.8 | 7.6 | 6.27 | 0.75 | 6.19 | 5.89 | A |
| F12 | 5.2 | 5.5 | 6.4 | 6.7 | 7.5 | 6.26 | 0.83 | 6.21 | 5.91 | A |
| F13 | 5.2 | 5.3 | 6.4 | 6.7 | 7.5 | 6.22 | 0.83 | 6.16 | 5.86 | A |
| F14 | 5.1 | 5.6 | 6.4 | 6.7 | 7.5 | 6.23 | 0.83 | 6.19 | 5.89 | A |
| F15 | 5.2 | 5.2 | 6.4 | 6.7 | 7.4 | 6.19 | 0.83 | 6.13 | 5.83 | A |
| F16 | 5.3 | 5.1 | 6.4 | 6.7 | 7.5 | 6.22 | 0.75 | 6.14 | 5.84 | A |

**Table S4. The mean plasma concentration (ug/mL) vs time (mins) profile data of 60 mg loxoprofen Na immediate release tablet in 24 healthy male Korean volunteers.**

| **Time** | **Time** | **Plasma Concentration** |
| --- | --- | --- |
| **T (min)** | **T (h)** | **Cp (ug/ml)** |
| 0.3834 | 0.00639 | 0.03226 |
| 8.9832 | 0.14972 | 1.13495 |
| 18.72517 | 0.312086 | 3.48025 |
| 28.87025 | 0.481171 | 4.48655 |
| 44.4574 | 0.740957 | 3.94578 |
| 59.43987 | 0.990665 | 2.85836 |
| 90.34542 | 1.505757 | 1.86264 |
| 90.34542 | 1.505757 | 1.86264 |
| 121.8557 | 2.030928 | 1.32187 |
| 180.9121 | 3.015202 | 0.81402 |
| 241.8498 | 4.03083 | 0.44959 |
| 359.8956 | 5.99826 | 0.10162 |

**Table S5.** Predicted vs Observed values of the pharmacokinetic parameters of optimized F2 formulation (120 mg) against IR tablet (60 mg).

| **Parameter** | **Observed (60 mg)** | **Expected (120 mg)** | **F2** | **FE** | **%PE** |
| --- | --- | --- | --- | --- | --- |
| *C_max_ (µg/ml)* | 4.4866 | - | 1.0958 | - | - |
| *T_max_ (h)* | 0.4812 | - | 4.8 | - | - |
| *AUC _0 to inf_ (µg-h/ml)* | 7.498 | 14.996 | 15.459 | 1.030875 | -3.09 |
| *AUC _0 to t_ (µg-h/ml)* | 7.364 | 14.728 | 14.448 | 0.980989 | 1.90 |

**Table S6.** Accelerated stability studies and shelf life of the optimized formulation (F2) as per ICH guidelines

| Study duration | **Parameters** | **Batch 1** | **Batch 2** | **Batch 3** |
| --- | --- | --- | --- | --- |
| 0 month | Physical appearance | No change observed | No change observed | No change observed |
|  | Hardness (Kg) | 1.21 ±0.02 | 1.25 ± 0.04 | 1.23 ±0.02 |
|  | Friability (%) | 0.61 ± 0.07 | 0.61 ± 0.07 | 0.61 ±0.07 |
|  | Floating lag time (s) | 35 ± 1.52 | 38 ±0.76 | 32 ±1.34 |
|  | Total lag time (h) | 21.5 ± 0.67 | 21 ±0.95 | 22 ±0.82 |
|  | Drug release (%) | 99.91 | 100.2 | 100.15 |
|  | Content uniformity (%) | 99.0 ±2.05 | 101 ±3.93 | 100 ±1.82 |
| 3 months | Physical changes | No change observed | No change observed | No change observed |
|  | Hardness (Kg) | 1.12 ±0.02 | 1.21 ±0.03 | 1.19 ±0.03 |
|  | Friability (%) | 0.61 ±0.07 | 0.61 ±0.07 | 0.61 ±0.07 |
|  | Floating lag time (s) | 37 ±1.9 | 41 ±1.3 | 36 ±1.1 |
|  | Total lag time (h) | 21.3 ±0.67 | 21.5 ±0.87 | 22.2 ±0.44 |
|  | Drug release (%) | 98.52 | 99.96 | 98.81 |
|  | Content uniformity (%) | 97.5±1.61 | 99.0 ±1.74 | 98.0 ±1.74 |
| 6 months | Physical changes | No change observed | No change observed | No change observed |
|  | Hardness (Kg) | 1.11 ±0.05 | 1.15 ±0.06 | 1.18 ±0.04 |
|  | Friability (%) | 0.61 ±0.07 | 0.61 ±0.07 | 0.61 ±0.07 |
|  | Floating lag time (sec) | 34 ±1.21 | 35 ±1.6 | 37 ±0.95 |
|  | Total lag time (h) | 21.3±0.56 | 21.8±0.57 | 21.2 ±0.97 |
|  | Drug release (%) | 99.23 | 99.26 | 99.19 |
|  | Content uniformity (%) | 101.23±1.61 | 98.0 ±1.74 | 96.0 ±1.74 |
| Shelf life (months) |  | 25.12 | 25.12 | 25.12 |

**Supplementary figures**

**
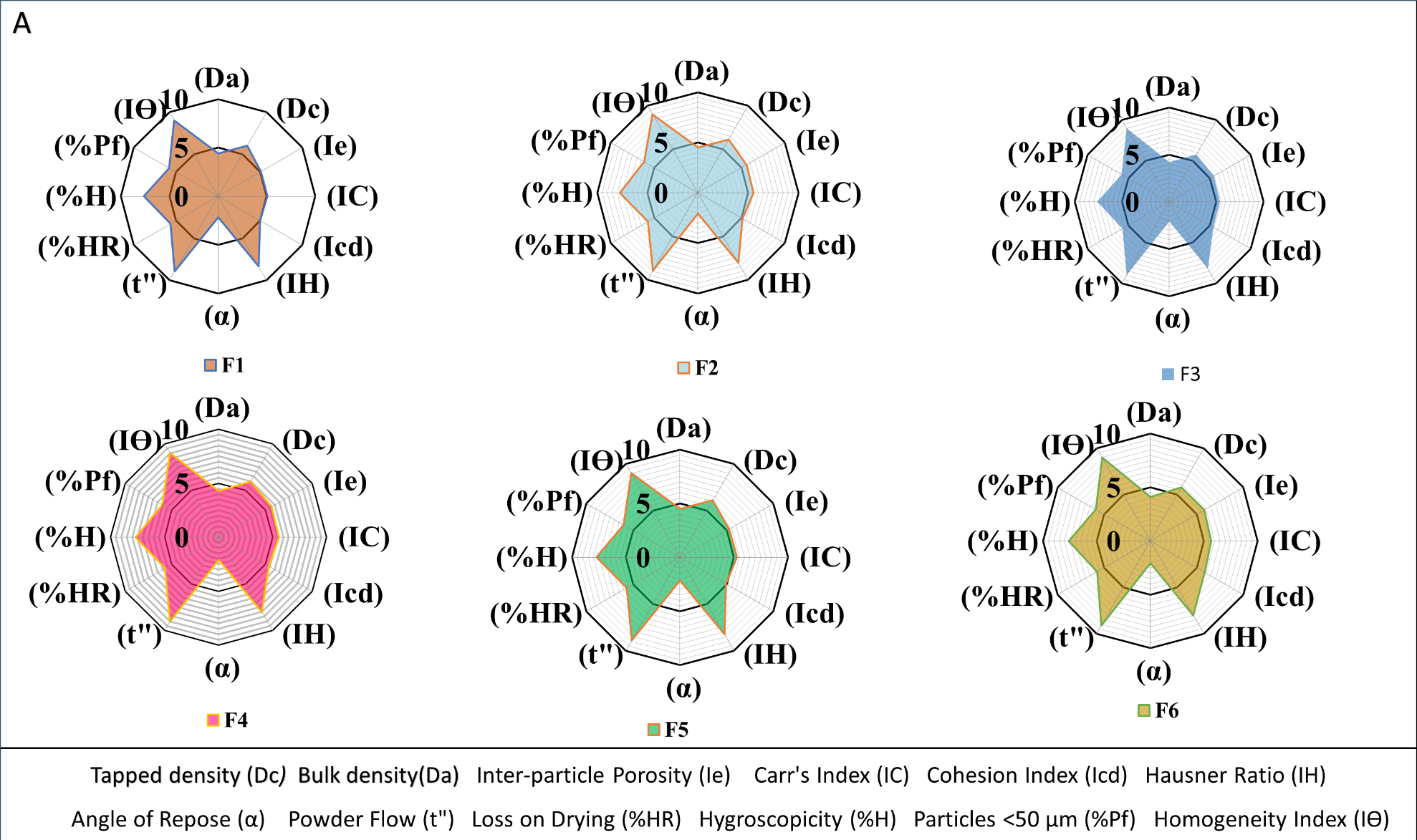
**

**Figure S1:** SeDeM diagram (radar graph) of formulations blends (A) F1-F6


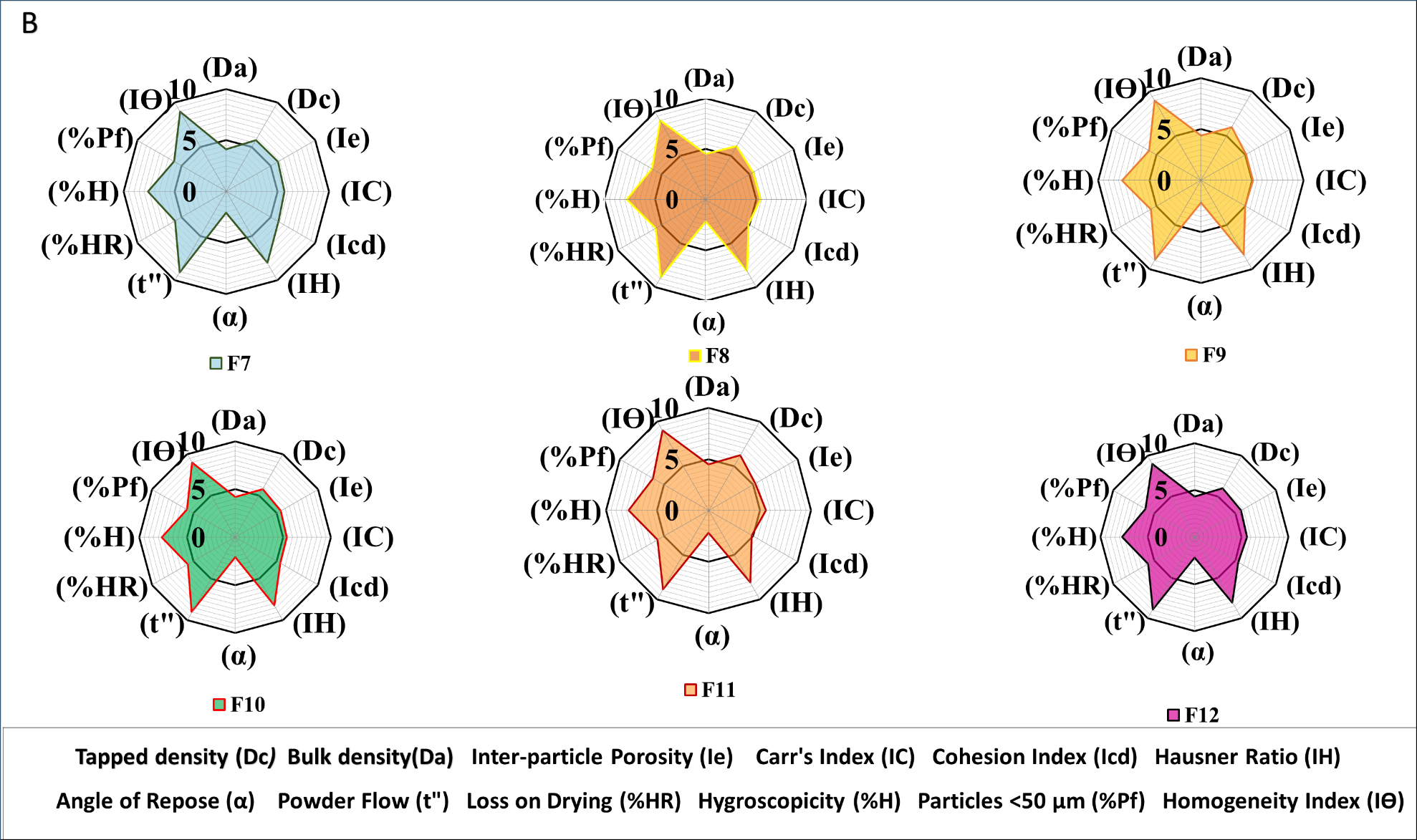


**Figure S2.** SeDeM diagram (radar graph) of formulations blends (B) F7-F12


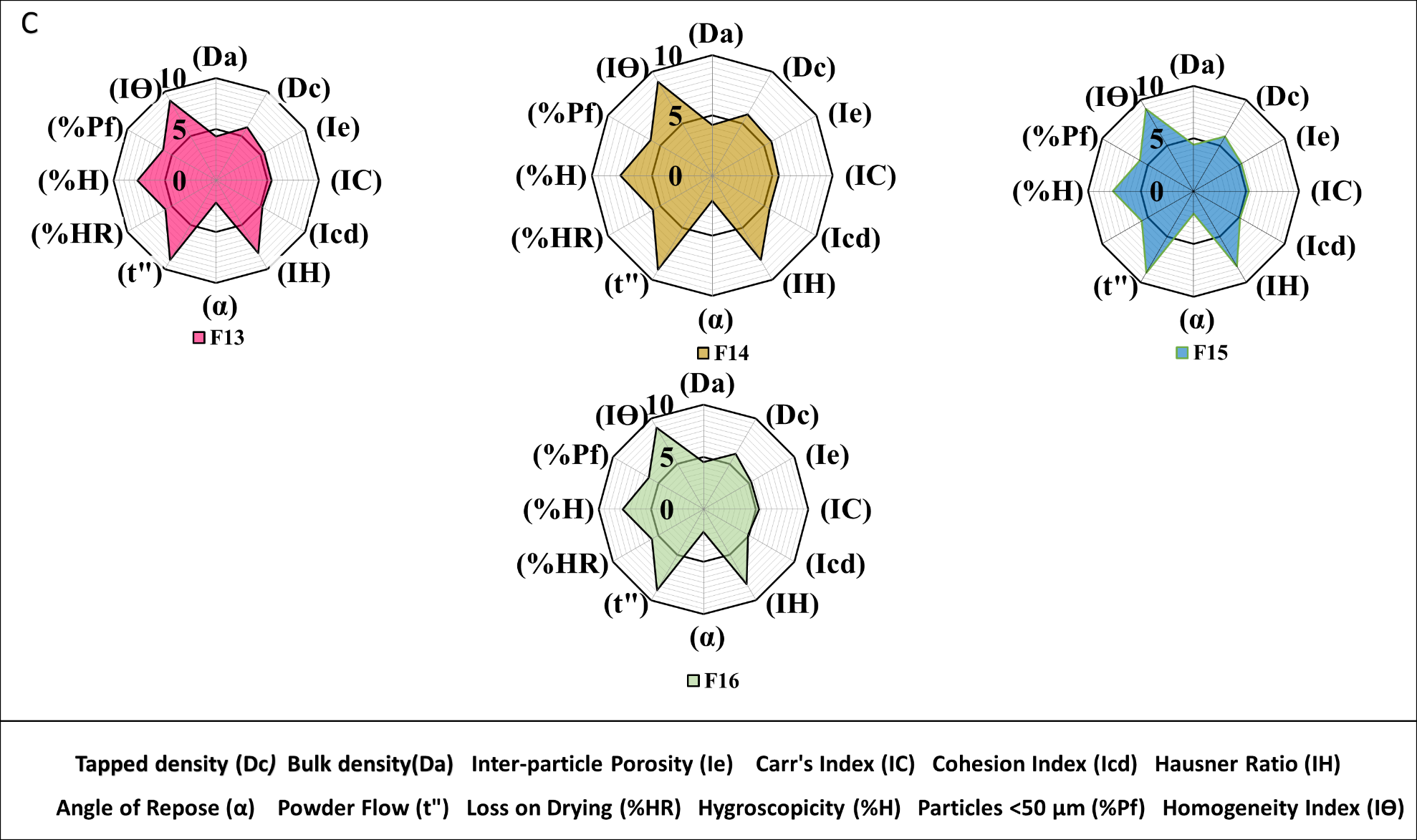


**Figure S3:** SeDeM diagram (radar graph) of formulations blends (C) F13-F16.


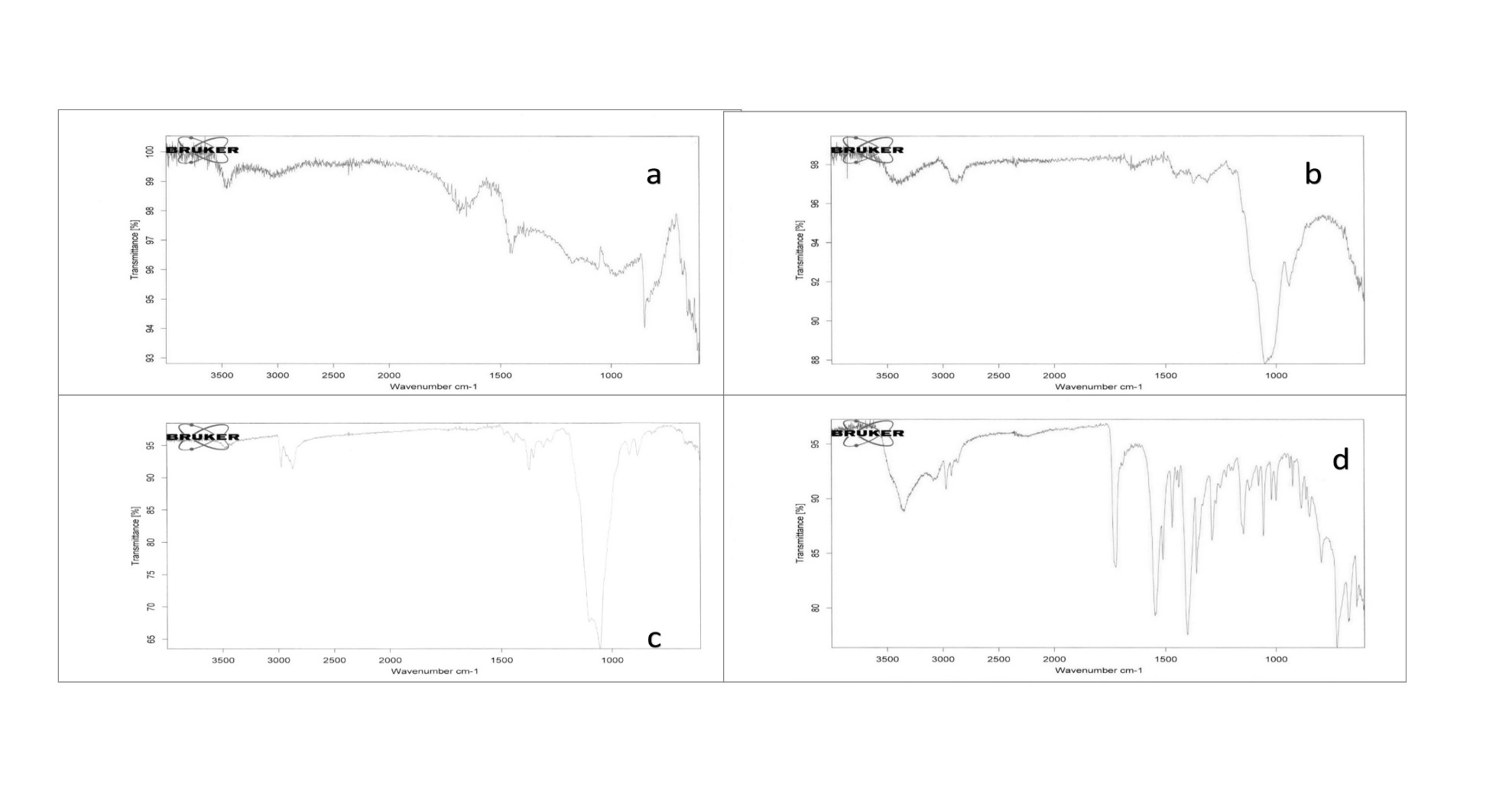


**Figure S4.** FTIR spectra of (a) sodium bicarbonate ( NaHCO_3_) (b) methocel K100M (c) EC-10 premium (d) loxoprofen sodium pure drug.


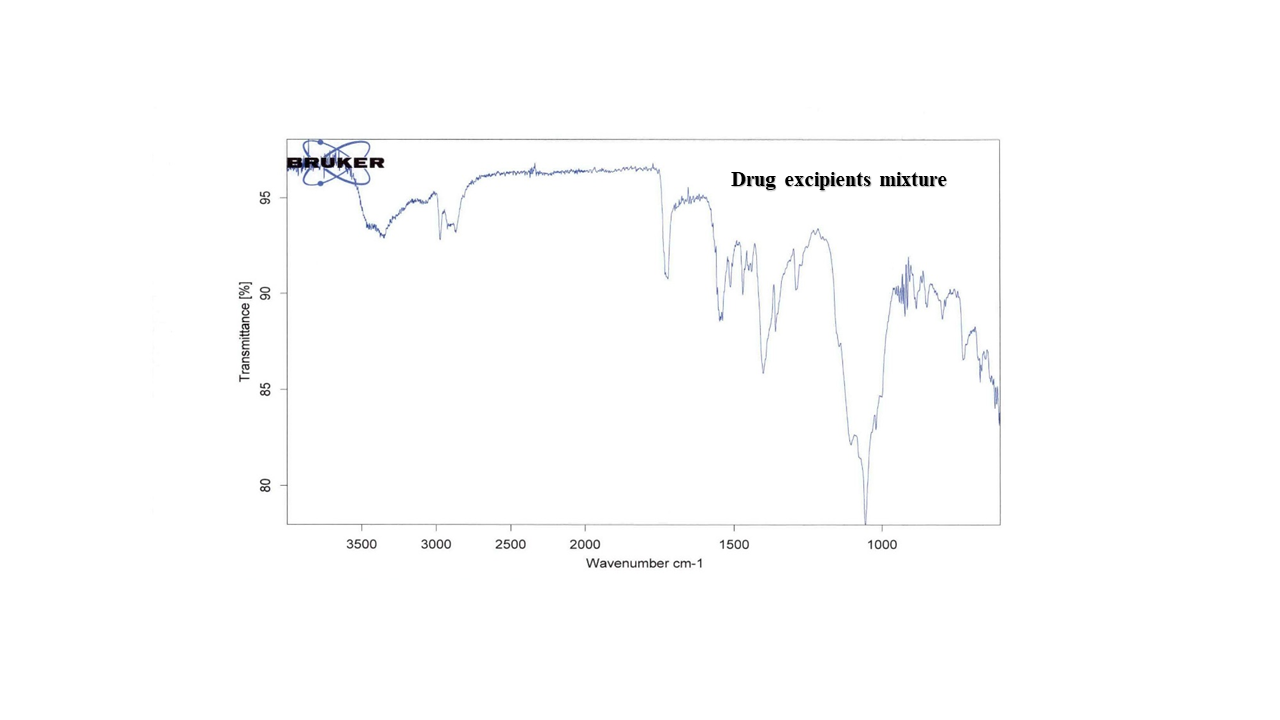
**Figure S5.** FTIR spectra of drug-excipients mixture of gastroretentive floating loxoprofen sodium mini-tablets.


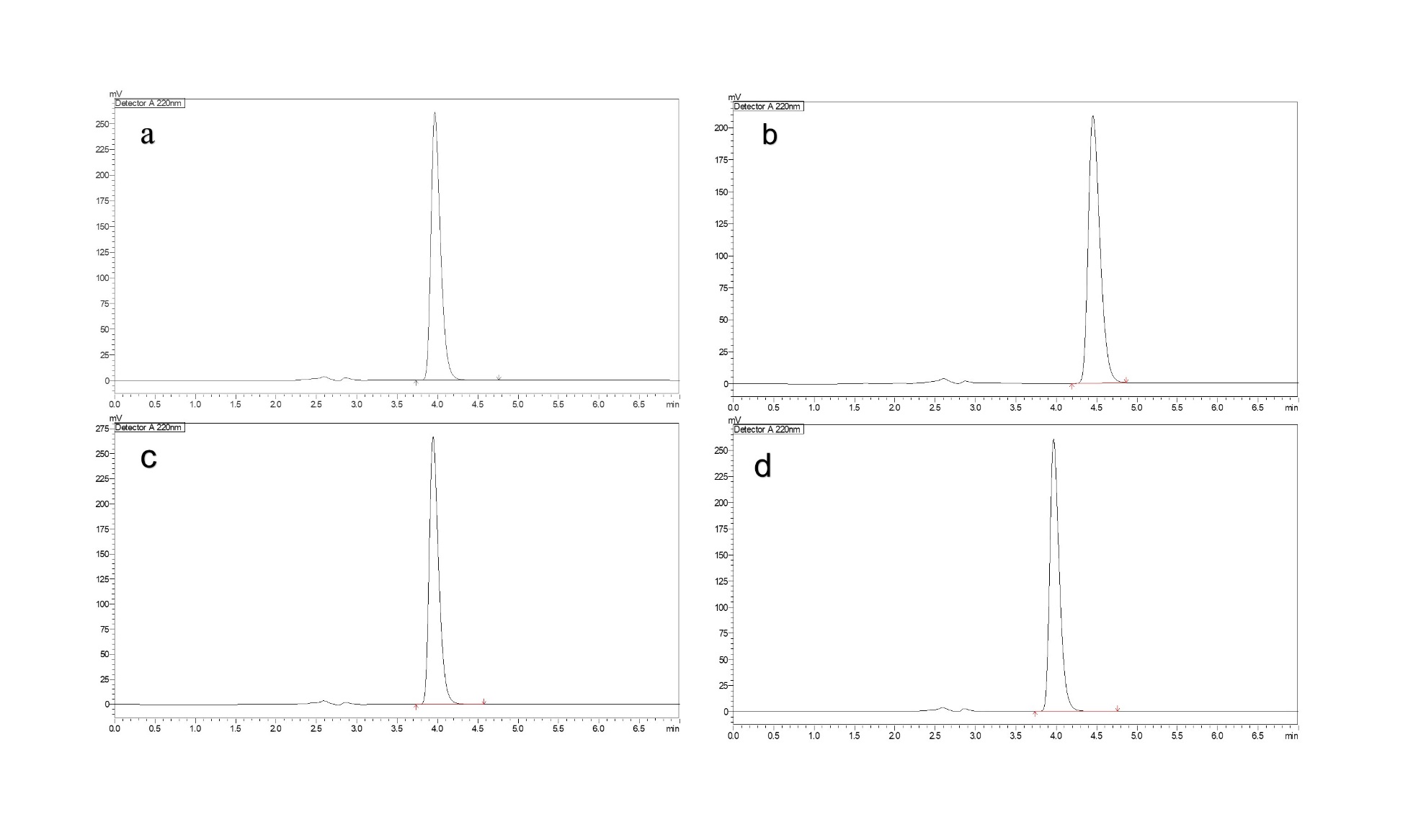
**Figure S6:** Chromatograms in mobile phase of (a) Loxoprofen sodium (LXP) (b) Loxoprofen sodium (LXP) (c) optimized formulation F2 (d) optimized formulation F2.
